# A satellite DNA array barcodes chromosome 7 and regulates totipotency via ZFP819

**DOI:** 10.1126/sciadv.abp8085

**Published:** 2022-10-28

**Authors:** Liane P. Fernandes, Rocio Enriquez-Gasca, Poppy A. Gould, James H. Holt, Lucia Conde, Gabriela Ecco, Javier Herrero, Robert Gifford, Didier Trono, George Kassiotis, Helen M. Rowe

**Affiliations:** ^1^ Centre for Immunobiology, Blizard Institute, Queen Mary University of London, London E1 2AT, UK.; ^2^ Bill Lyons Informatics Centre, UCL Cancer Institute, London WC1E 6BT, UK.; ^3^ School of Life Sciences, Ecole Polytechnique Fédérale de Lausanne, Lausanne, Switzerland.; ^4^ MRC–University of Glasgow Centre for Virus Research, Glasgow, UK.; ^5^ The Francis Crick Institute, 1 Midland Road, London NW1 1AT, UK.

## Abstract

Mammalian genomes are a battleground for genetic conflict between repetitive elements and KRAB-zinc finger proteins (KZFPs). We asked whether KZFPs can regulate cell fate by using ZFP819, which targets a satellite DNA array, ZP3AR. ZP3AR coats megabase regions of chromosome 7 encompassing genes encoding ZSCAN4, a master transcription factor of totipotency. Depleting ZFP819 in mouse embryonic stem cells (mESCs) causes them to transition to a 2-cell (2C)–like state, whereby the ZP3AR array switches from a poised to an active enhancer state. This is accompanied by a global erosion of heterochromatin roadblocks, which we link to decreased SETDB1 stability. These events result in transcription of active LINE-1 elements and impaired differentiation. In summary, ZFP819 and TRIM28 partner up to close chromatin across *Zscan4*, to promote exit from totipotency. We propose that satellite DNAs may control developmental fate transitions by barcoding and switching off master transcription factor genes.

## Teaser

An array of satellite DNA repeats on chromosome 7 acts as a switch enhancer to regulate *Zscan4* genes and totipotency.

## INTRODUCTION

Mammalian genomes must maintain a fine balance between their constant evolution to permit adaptation, while safeguarding genome integrity (*1*). For example, the endogenous retrovirus (ERV) MERVL has been co-opted by the host to drive expression of genes expressed at the 2-cell (2C) stage of development (*2*–*4*), but ERVs are also selfish genome invaders that can spread and mutate host genomes (*5*). Thus, the necessary regulation of repetitive elements (REs) is achieved through their transcriptional silencing by host defense proteins known as krueppel-associated box (KRAB)–zinc finger proteins (KZFPs) (*6*–*8*). KZFPs are sequence specific, and early in development, they home in to target RE sequences and recruit KRAB-associated protein 1 (TRIM28) and downstream enzymes, including Set Domain Bifurcated histone lysine methyltransferase 1 (SETDB1), to initiate site-specific heterochromatin (*9*–*11*). While some KZFPs have known roles in RE repression (*8*, *12*–*16*), others have been shown to instead target and regulate host genes (*17*–*19*), although still little is known about the dialog between KZFPs and their host genomes. Here, we hypothesize that regulatory networks of REs and their cognate KZFPs, which first evolved to restrict RE spread, may have been co-opted by the host to control developmental fate transitions.

## RESULTS

### Cells undergo a 2C-like fate transition upon loss of ZFP819

**Fig. 1.**
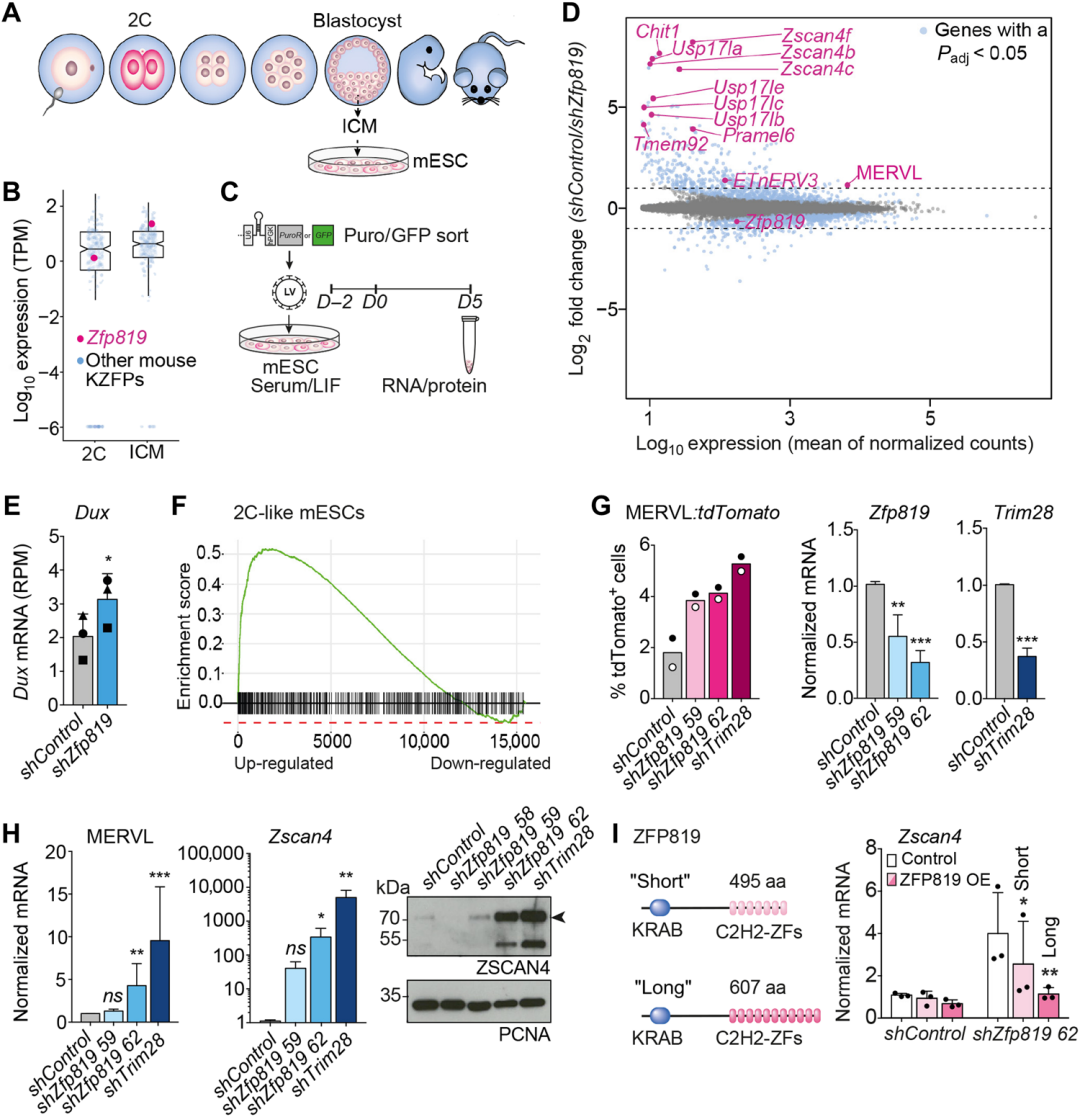
mESCs transition to a 2C-like state upon loss of ZFP819. (**A**) mESCs are used to model reprogramming to a state with similarities to 2C embryos. ICM, inner cell mass; 2C, 2-cell. (**B**) Transcript levels of 327 KZFPs, including *Zfp819*, in a 2C embryo and the blastocyst. KZFPs, KRAB zinc finger proteins; TPM, transcripts per million. (**C**) Experimental setup: shRNAs are added on day −2. LIF, leukemia inhibitory factor. (**D**) Differentially expressed genes and transposable element (TE) families upon ZFP819 depletion, compared to control. Events beyond the dashed lines represent log_2_ fold changes >1 or < −1. Highlighted in blue are genes with a *P*-adjusted value < 0.05. 2C genes are labeled in pink. (**E**) RNA-seq reads mapping to *Dux* in control and ZFP819-depleted cells. *P* = 0.0451 (two-tailed paired Student’s *t* test). RPM, reads per million. (**F**) Gene set enrichment analysis of 2C-like genes (*3*). *P*-adjusted value = 0.0002. (**G**) Left: MERVL-tdTOMATO–positive cells, following ZFP819 depletion (mean of two independent experiments). Right: RT-qPCR analysis of *Zfp819* and *Trim28* transcripts, following their shRNA depletion (mean ± SD of three independent experiments). *P* values: 0.0090 (*shZfp819 59*), 0.0005 (*shZfp819 62*), and 0.0005 (*shTrim28*) (two-tailed paired Student’s *t* tests). (**H**) RT-qPCR validation of 2C genes up-regulated (left) and Western blot of ZSCAN4 protein (right). One representative experiment of three is shown. RT-qPCR data show mean ± SD of three independent experiments. *P* values for MERVL: 0.0093 (*shZfp819 62*) and 0.0008 (*shTrim28*). *P* values for *Zscan4:* 0.0415 (*shZfp819 62*) and 0.0022 (*shTrim28*) (two-tailed unpaired Student’s *t* tests). ns, not significant. (**I**) Domains of ZFP819 Short and Long isoforms (left). RT-qPCR detection of *Zscan4* (right): Rescue experiments were performed with non–HA-tagged constructs. Data are mean ± SD of three independent experiments with replicate values shown. *P* values: short isoform = 0.0357; long isoform = 0.0011 (two-tailed paired Student’s *t* tests). aa, amino acids.

We explored whether REs and their cognate KZFPs control developmental gene networks using mouse embryonic stem cells (mESCs) (Fig. 1A) and the mouse KZFP, ZFP819, which has been implicated in pluripotency (*20*). *Zfp819* is one of the most highly expressed KZFP mRNAs at the blastocyst stage, yet it is relatively lowly expressed at the 2C stage compared to other KZFPs (Fig. 1B and fig. S1A) (*21*). Targeting *Zfp819* for short hairpin RNA (shRNA)–mediated depletion in mESCs (Fig. 1C and fig. S1B) to mimic its lower pattern of expression at earlier stages of development was sufficient to induce a transition of mESCs to resemble a 2C-like state, as measured by RNA sequencing (RNA-seq) (Fig. 1D and data file S1). Classical 2C genes and ERVs were up-regulated including *Zscan4*, *Dux*, MERVL, and a cluster of genes encoding the USP17 deubiquitinating enzymes (Fig. 1, D and E and fig. S1C) (*2*, *22*–*28*). Gene set enrichment analyses further confirmed that ZFP819-depleted mESCs adopt a 2C fate (Fig. 1F and fig. S1DE). Concordantly, using MERVL-t*dTomato* reporter mESCs, which can be used to identify rare mESCs existing in a 2C-like state (*2*, *3*), we confirmed that ZFP819 depletion leads to an increase in tdTOMATO-positive mESCs, similar to that seen for TRIM28 depletion. This suggests that ZFP819 may be one of the main KZFPs with which TRIM28 participates to regulate developmental potency (Fig. 1G) (*3*). We verified up-regulation of 2C genes in ZFP819-depleted mESCs using reverse transcription quantitative polymerase chain reaction (RT-qPCR). By Western blot, we detected a decrease in protein levels of POU Domain, Class 5 Homeobox 1 (POU5F1/OCT4) as expected (*3*) and a notable overexpression of Zinc finger and SCAN domain containing 4 (ZSCAN4) (Fig. 1H and fig. S2AB). This 2C shift was evident using either puromycin selection or green fluorescent protein (GFP) sorting of ZFP819-depleted cells and was recapitulated in mESCs from different mouse strains (fig. S2, C to F). Last, we could rescue this cell fate transition by overexpressing the canonical full-length isoform of ZFP819 encompassing 11 zinc fingers (ZFs) (Fig. 1I and fig. S2G). Significant rescue was also achieved by overexpression of a shorter, unannotated isoform of ZFP819 that harbors the first seven ZFs and which we found to also be expressed in mESCs (Fig. 1I and fig. S2G).

### ZFP819 targets a satellite repeat, ZP3AR, coating chromosomes 5 and 7

**Fig. 2.**
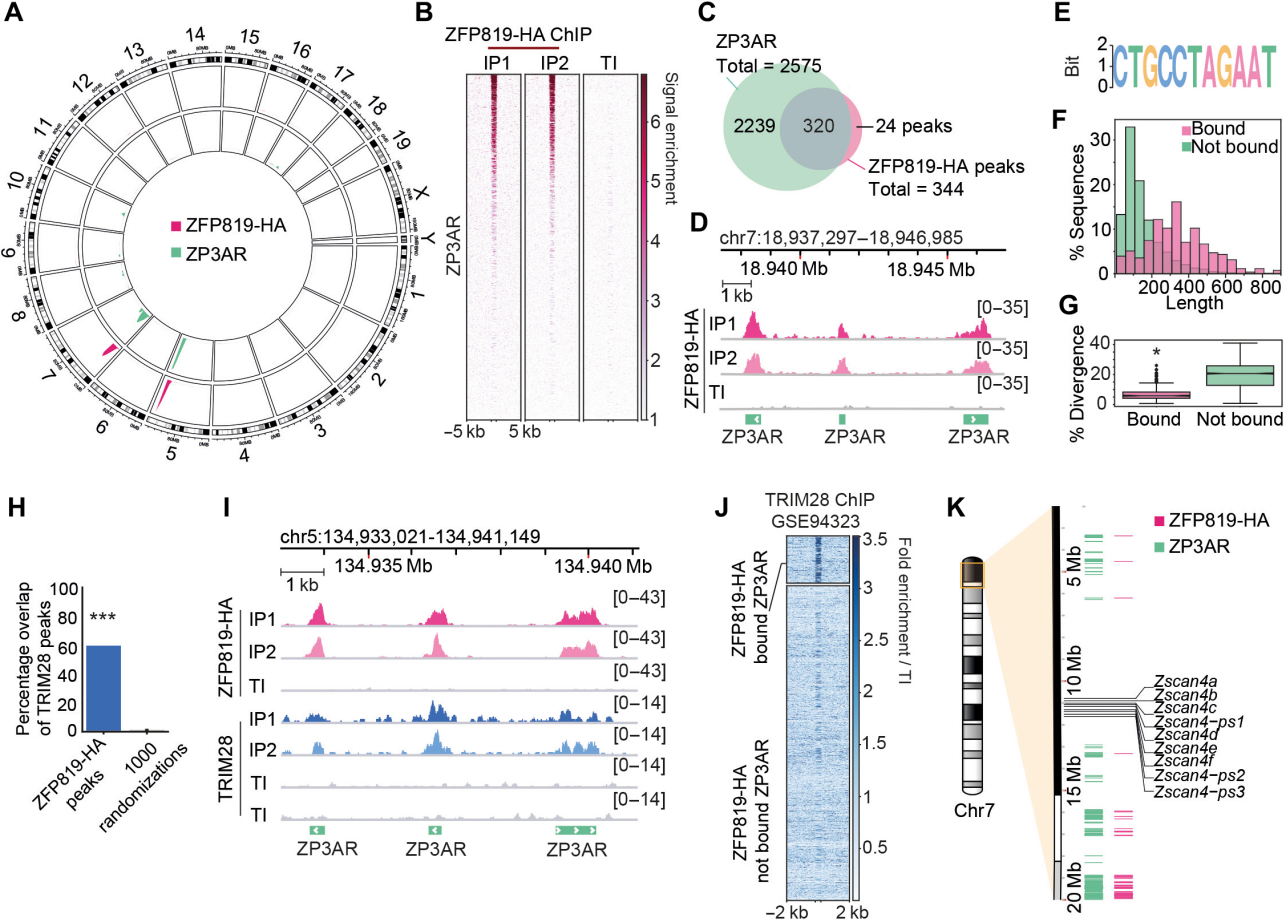
ZFP819 targets a satellite repeat, ZP3AR, coating chromosomes 5 and 7. (**A**) ZFP819-HA ChIP peaks and ZP3AR repeat densities illustrated on a circos plot of genome visualization. HA, hemagglutinin. (**B**) Heatmap of ZFP819-HA ChIP normalized read coverage at ZP3ARs sorted by ZFP819-HA signal intensity. Duplicate IPs and TI (total input) control are shown. (**C**) Venn diagram representing the overlap of ZFP819-HA MACS2-called peaks and ZP3AR instances. (**D**) Example genome track views of ZFP819-HA ChIP-normalized read coverage at several ZP3AR instances. (**E**) Motif logo generated using ZFP819-HA ChIP peak sequences. *E* = 5^−3312^, where the *E* value for the motif is an estimate of the number of such motifs expected in shuffled input sequences, using Multiple Em for Motif Elicitation (MEME). (**F**) Histogram of ZP3AR sequence lengths separated by ZFP819-HA binding, as determined in (C). (**G**) Percentage divergence of ZP3ARs, from ZP3AR consensus, separated into ZFP819-HA–bound (336 loci) and not bound ZP3ARs (2239 loci). *P* = 3.8 ×10^−153^ (two-tailed Student’s *t* test). (**H**) Percentage overlap of ZFP819-HA peaks with published TRIM28 ChIP-seq data (*23*). A total of 1000 randomizations were used to assess significance. *P* = 0.000999, where a one-sided *P* value was calculated as 1+ number of randomizations with an overlap greater than the observed value/1000 randomizations. (**I**) Genome track view of ZFP819-HA ChIP and public TRIM28 ChIP (*23*) normalized read coverage at example ZP3ARs. (**J**) TRIM28 ChIP signal normalized to total input (TI) at ZFP819-HA-bound versus not bound ZP3ARs, sorted by ZFP819-HA binding intensity. (**K**) Karyotype plot annotated with coordinates of ZFP819-HA ChIP peaks, ZP3ARs, and the *Zscan4* gene cluster.

To identify the binding profile of ZFP819 in mESCs, we performed chromatin immunoprecipitation (ChIP) of full-length, hemagglutinin (HA)–tagged ZFP819, and we used ZFP809-HA, which is known to bind endogenous MLVs (*8*, *29*), as a control (fig. S3A). ZFP819 exhibited remarkable specificity for a satellite repeat, ZP3AR, clustered across megabase regions of chromosomes 5 and 7, while ZFP809 bound to endogenous MLV targets, as expected (Fig. 2, A to D, and fig. S3BC). Inspection of ZFP819-bound ZP3ARs revealed that ZP3AR is composed of multiple copies of a short interspersed element (SINE) of the ID4 type embedded within a repeated sequence (fig. S3D). ZFP819 recognizes a sequence containing a motif at the junction of the SINE element and adjacent repeated sequence (Fig. 2E and fig. S3D). Notably, ZFP819-bound ZP3ARs, which are restricted to chromosomes 5 and 7, are longer than unbound copies and less diverged in sequence (Fig. 2, F and G), with respect to the consensus. We found that there is significant overlap between TRIM28 peaks and ZFP819-bound sites (Fig. 2, H to J, and fig. S3E), suggesting that ZFP819 may restrict the expansion of ZP3ARs by partnering with TRIM28 and SETDB1 to initiate heterochromatin at these repeats. Satellite DNA expansions can result from replication errors and potentially from RNA-driven reverse transcription and reintegration (*30*), threatening genome stability. ZP3ARs and ZFP819 are conserved throughout six rodent families including rats and older rodent species (fig. S4), illustrating that their partnership may represent a key gene-regulatory pathway preserved for more than 12 million years. Owing to the *Zscan4* gene cluster being embedded within the chromosome 7 ZP3AR array, we reasoned that ZP3AR may control *Zscan4* gene expression through cis-regulation (Fig. 2K).

### ZFP819 depletion induces a global erosion of H3K9me3

**Fig. 3.**
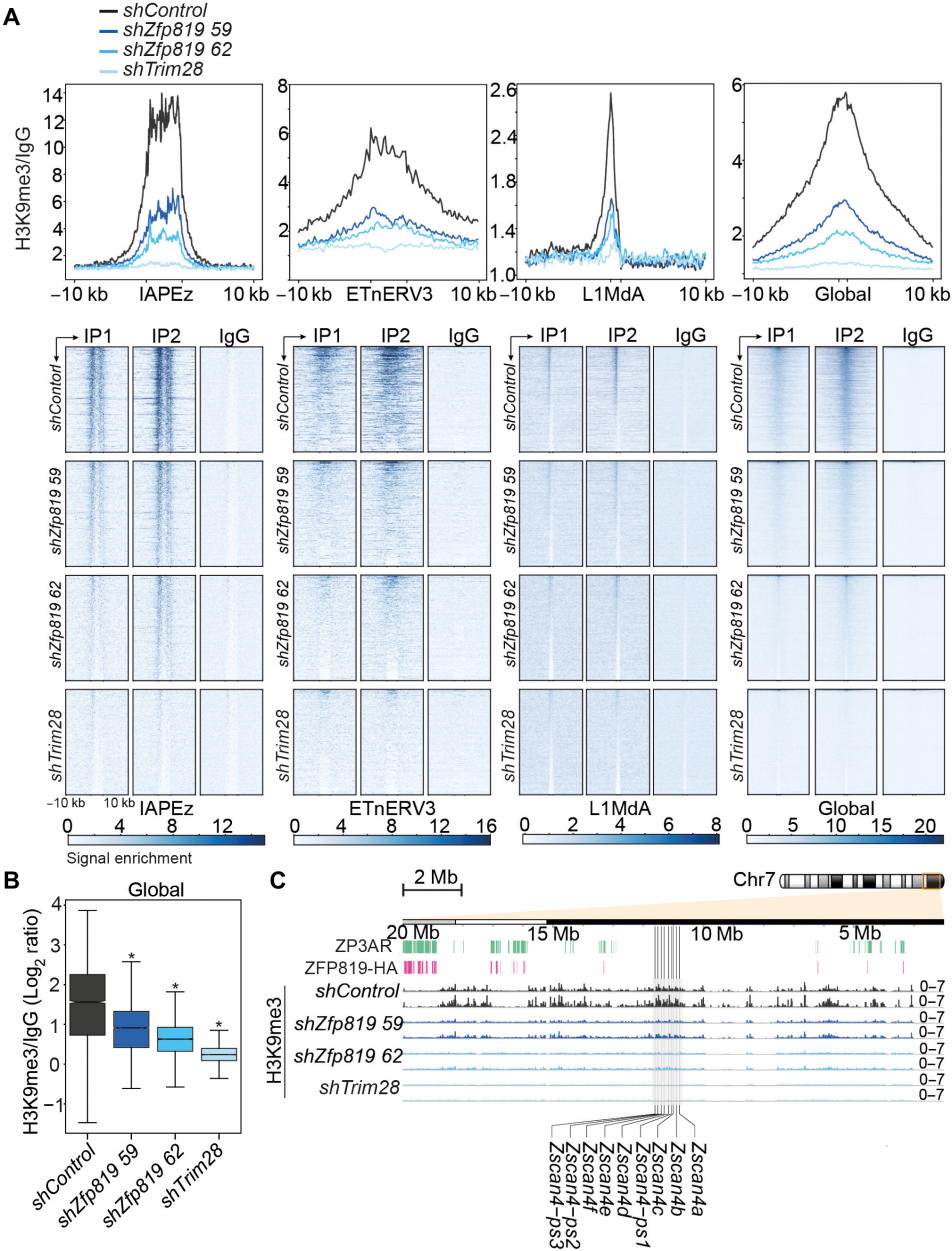
ZFP819 depletion induces a global erosion of H3K9me3. (**A**) Profile plots, with a 10-kb flank, represent the trimmed mean of H3K9me3 CUT&RUN coverage normalized to IgG at repeats and global H3K9me3 enrichment sites (top). Heatmaps of H3K9me3 CUT&RUN normalized read coverage, with a 10-kb flank, at repeats and global H3K9me3 sites (bottom). H3K9me3 CUT&RUN profiling was performed in GFP^bright^ sorted *shControl*, *shZfp819*, and *shTrim28* mESCs. The top 5000 H3K9me3 peaks obtained from the mouse ENCODE project define global H3K9me3 enrichment sites. (**B**) Distribution of the mean H3K9me3 signal at global H3K9me3 enrichment sites (top 5000 ENCODE peaks) for *shControl*, *shZfp819*, and *shTrim28* mESCs. *P* = 0.0 (*shZfp819 59, shZfp819 62*, and *shTrim28*) (two-tailed paired Student’s *t* test compared to *shControl*). (**C**) Genome track view of an 18-Mb region along chromosome 7. H3K9me3 is represented as fold enrichment (H3K9me3 CUT&RUN normalized to IgG) in *shControl*, *shZfp819*, and *shTrim28* mESCs. Coordinates of ZFP819-HA ChIP peaks, ZP3AR, and the *Zscan4* gene cluster are annotated.

**Fig. 4.**
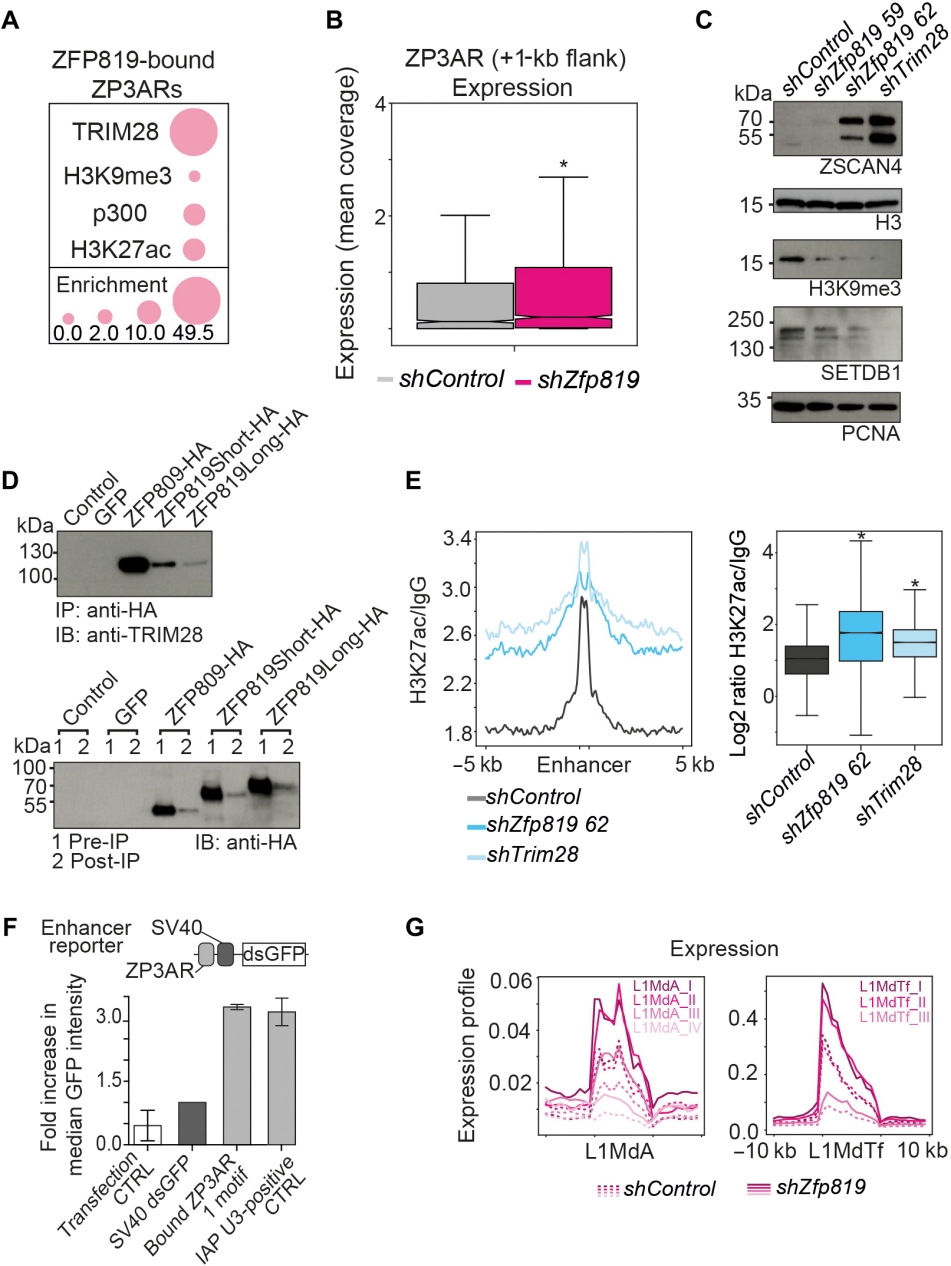
Chromatin remodeling is through activation of ZP3AR enhancers and degradation of SETDB1 protein. (**A**) Observed over expected enrichment of the stated chromatin features at ZFP819-HA–bound ZP3ARs, with enrichment values given. *P* values were assessed using 1000 randomizations and were <0.05 for all associations, except H3K9me3, due to its low level but this reached significance in multiple datasets (fig. S5B). (**B**) Mean ZP3AR expression signal (across 2575 loci). *P* = 2.364 × 10^−7^ (two-tailed paired Student’s *t* test). (**C**) Western blot of ZSCAN4, H3K9me3, and SETDB1. Histone H3 and Proliferating Cell Nuclear Antigen (PCNA) were loading controls. One representative experiment of two is shown. (**D**) Coimmunoprecipitation of TRIM28 with HA-tagged ZFP809, ZFP819Short, and ZFP819Long (left) in HEK293T cells. Western blot of HA-tagged ZFP809, ZFP819Short, and ZFP819Long pre- and post-IP (right) in HEK293T cells. (**E**) Profile plot, with a 5-kb flank of the trimmed mean of H3K27ac CUT&RUN coverage normalized to IgG at ENCODE enhancers (across 12, 142 peaks) in *shControl*, *shZfp819*, and *shTrim28* mESCs (left). Distribution of the mean H3K27ac signal at enhancers from the left dataset (right). *P* = 0.0 (*shZfp819 59*, *shZfp819 62*, and *shTrim28*) (two-tailed paired Student’s *t* tests). (**F**) A ZFP819-bound ZP3AR (chr7:19741609-19741700) was cloned upstream of a minimal SV40 promoter driving destabilized GFP (dsGFP). ZP3AR enhancer activity was measured in NIH/3T3 cells 2 days after transfection. The SV40-dsGFP backbone alone was a negative control, and the positive control was the U3 region of an intracisternal A-type particle (IAP) element that we recently found to act as an enhancer (*62*). (**G**) Expression signal of L1Md_A subfamilies (top) and L1Md_T subfamilies (bottom) in ZFP819-depleted mESCs using *shZfp819* hairpin 62. L1Md_A *P* values: 0.3548 (I), 0.3184 (II), 1.9875 × 10^−11^ (III), and 0.0359 (IV). L1Md_T *P* values: 5.1696 × 10^−70^ (I), 0.0003 (II), and 0.004 (III) (two-tailed paired Student’s *t* tests).

We next used CUT&RUN chromatin profiling to measure H3K9me3 levels, because KZFPs and TRIM28 are known to recruit SETDB1 to deposit silent H3K9me3 at their retroelement target sites (*12*, *29*). Unexpectedly, global H3K9me3 levels, which are mainly enriched at intracisternal A-type particle (IAPEz) elements, decreased upon loss of ZFP819 (Fig. 3A). Heterochromatin not only silences repeats but also provides structure and preserves cell identity and transcriptional integrity (*31*). We therefore refer to this decrease in global H3K9me3 as a loss of heterochromatin “roadblocks” as reference to the broad implications this may have on the cell. H3K9me3 was also decreased at active early transposon (ETn) endogenous retroviruses and young Long INterspersed Element-1 (LINE-1) elements (L1Md_A) (Fig. 3, A and B), which are not direct targets of ZFP819 (fig. S5A). H3K9me3 levels also dropped at the *Zscan4* gene cluster region, explaining the induction of ZSCAN4 expression (Fig. 3C). Only ZP3AR copies that were bound by ZFP819 exhibited a significant H3K9me3 signature in mESCs (fig. S5BC), suggesting that these satellite DNAs may represent enhancers that get switched to a poised state via ZFP819. To address this, we assessed the chromatin state of ZP3ARs bound by ZFP819 using Encyclopedia of DNA Elements (ENCODE) data from mESCs. With an enrichment of active enhancer signatures (H3K27ac and p300) and repressive chromatin (TRIM28 and H3K9me3), these sites resembled poised enhancers regulated by H3K9me3 (Fig. 4A and fig. S5, B and C) (*31*). Consistent with this, ZFP819 depletion resulted in an increase in ZP3AR RNAs, which may, therefore, function as enhancer RNAs (Fig. 4B) (*32*).

### An instability in SETDB1 protein levels is linked to chromatin remodeling events

Considering that loss of ZFP819 also leads to the global erosion of heterochromatin roadblocks (Fig. 3), we asked whether protein stability of SETDB1 was affected in ZFP819-depleted cells. Western blots showed that ZFP819 depletion results in a loss of SETDB1 protein levels and global H3K9me3, almost to the same extent as TRIM28 inactivation (Fig. 4C). SETDB1 is targeted for proteasomal degradation unless it is retained in the nucleus (*33*, *34*) where it is monoubiquitinated, which is essential to its function (*35*, *36*). We hypothesized that a ZFP819-TRIM28 complex might contribute to nuclear retention of SETDB1 levels, and we validated that ZFP819 interacts with TRIM28 by coimmunoprecipitation (Fig. 4D). However, indirect phenomena, which the loss of ZFP819 precipitates, such as the increased expression of 2C genes, might also promote degradation of SETDB1. Unexpectedly, other histone methyltransferase (HMT) enzymes (*37*) are not able to compensate for the loss of SETDB1 here, implying that the reduction of global heterochromatin may mirror an earlier embryonic-like state where H3K9me3 is naturally low.

Consistent with the loss of SETDB1, we detected an increase in H3K27ac levels at genome-wide enhancers (Fig. 4E) (*38*). Furthermore, we could demonstrate that a ZFP819-bound ZP3AR functions as a bona fide enhancer using a reporter assay in NIH/3T3 mouse fibroblasts, which lack expression of ZFP819 (Fig. 4F). Of interest, we found the ZP3AR consensus sequence to contain repeated motifs for the reprogramming factor PRDM14 (fig. S5D), which may use ZP3ARs to remodel cell fate. With this epigenetic switch from the loss of heterochromatin roadblocks to the increase in global H3K27ac enrichment, following ZFP819 depletion, we asked whether repeats are also derepressed. We uncovered that there is a shift in transcriptional up-regulation of evolutionarily young LINE-1 elements specifically (Fig. 4G), which can contribute to chromatin accessibility (*39*). Indeed, some of these elements are still active for retrotransposition and linked to genome instability (*40*). IAPEz elements, in contrast, were not derepressed (fig. S5E), perhaps because they retained higher H3K9me3 levels than in TRIM28-depleted cells (Fig. 3). Consistent with these latter results, de novo enhancers were unveiled at young LINE-1 elements in the ZFP819-depleted cells, whereas in the TRIM28-depleted cells, enhancers were derepressed at both young LINE-1 elements and IAPEz elements (figs. S6, A and B, and S7, A to C). Notably, depletion of LINE-1 RNA has been shown to enhance a transition of mESCs to a 2C-like state (*28*), but this effect may relate to specific copies of co-opted LINE-1s not examined here. Last, analyses of public Assay for Transposase-Accessible Chromatin with sequencing (ATAC-seq) datasets through mouse development showed that ZFP819-bound ZP3ARs are enriched for chromatin accessibility at the 2C stage of mouse development (fig. S8A). At this developmental stage, the *Zscan4* cluster of genes has captured stretches of ZP3AR enhancers at either side of it within the same chromatin compartment, as revealed by using high-throughput chromosome conformation capture (HiC) data through mouse development (fig. S8B). A conformational switch by the 8C stage suggests that the *Zscan4* cluster no longer has access to these enhancers (fig. S8, B and C). These data, along with the gain of H3K9me3 at ZFP819-bound ZP3ARs (fig. S5, B and C), support a proposed model whereby the ZP3AR array functions as a switch enhancer to regulate the exit from totipotency.

### Impaired differentiation potential is linked to ZFP819 depletion

**Fig. 5.**
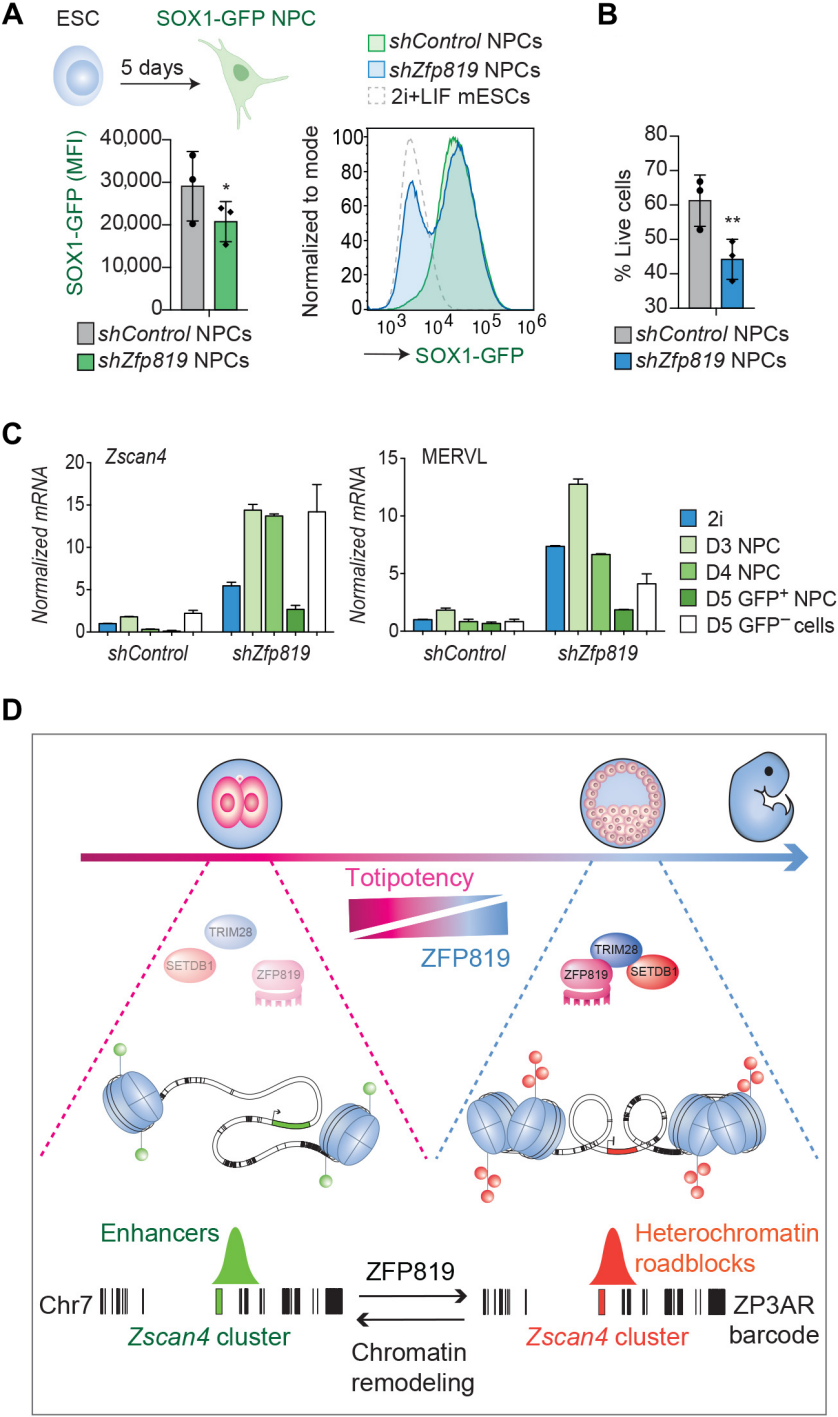
Impaired differentiation potential linked to ZFP819 depletion. (**A**) NPC differentiation of SOX1-GFP reporter mESCs treated with *shControl* or *shZfp819 62* constructs. Summary data of the GFP measurement for three independent experiments, after gating on live cells (left). *P* = 0.0187 (two-tailed paired Student’s *t* test). Representative histogram overlays shown for one of the three experiments (right). NPCs, neural progenitor cells; MFI, mean fluorescence intensity; LIF, leukemia inhibitory factor. Diagram created with BioRender.com. (**B**) Percentage of live cells following NPC differentiation of control and ZFP819-depleted mESCs, using *shZfp819* 62. *P* = 0.0018 (two-tailed paired *t* test). (**C**) RT-qPCR validation of expression of the 2C gene *Zscan4* and the 2C-stage endogenous retrovirus, MERVL up-regulated in day 3, day 4, and day 5 sorted GFP^+^ NPCs as well as in day 5 sorted GFP^−^ cells. See fig. S9 for *Pax6* data. 2i, two inhibitors (naïve state mESCs) (**D**) Proposed model. ZFP819 likely evolved to silence ZP3AR in early development, which led to its co-option by the host to facilitate exit from totipotency through cis-regulation of the *Zscan4* gene cluster. ZFP819 recruits TRIM28 and H3K9me3 to the ZP3AR enhancer array and stabilizes levels of SETDB1 to promote the instatement of heterochromatin roadblocks. While the satellite repeat ZP3AR barcodes chromosome 7, by analogy, other satellite DNAs may barcode other gene clusters, encoding stage-specific master transcription factors, like ZSCAN4. The chromosome 7 ZP3AR barcode here is to scale and exported from the UCSC genome browser repeatmasker ZP3AR track.

Reprogramming of cells to an open chromatin state can be induced using defined transcription factors (*41*) and is a hallmark of early embryos (*21*) and of cancer initiating cells that escape lineage commitment (*42*, *43*). We reasoned that ZFP819-inactivated mESCs that have lost heterochromatin roadblocks may exhibit impaired differentiation. To test this hypothesis, we set up an in vitro assay of ESC to neural progenitor cell (NPC) differentiation using SRY-box transcription factor 1 (SOX1)–GFP reporter mESCs. When gating on live cells, we found that ZFP819-inactivated mESCs showed a decreased potential to differentiate into NPCs (Fig. 5A). In addition, ZFP819-depleted cells displayed reduced viability (Fig. 5B). To explore the basis for this differentiation defect further, we sorted the SOX1-GFP–positive NPCs from the control and ZFP819-depleted groups and assessed expression of the neural marker, *Pax6*, and 2C genes by RT-qPCR (Fig. 5C and fig. S9). The ZFP819-depleted NPCs expressed equivalent levels of *Pax6* to control NPCs (fig. S9AB), but they exhibited unwarranted activation of MERVL and *Zscan4* (Fig. 5C and fig. S9C). These data suggest that a block in differentiation is likely not due to a failure to activate the neural transcriptional program but due to a decreased ability to exit the 2C program (fig. S9D). In summary, we propose a model where a ZFP819-TRIM28-SETDB1 axis facilitates the exit of ZSCAN4-mediated totipotency and the global instatement of heterochromatin roadblocks (Fig. 5D).

## DISCUSSION

These results suggest that while ZFP819 evolved to bind to and restrict the spread of ZP3AR satellite repeats, this interaction now helps to switch off totipotency through cis-regulation of the *Zscan4* gene cluster. Inactivation of ZFP819 leads to the erosion of heterochromatin roadblocks, the foci of which reside mainly at IAPEz elements. This results in a genome-wide enhancer switch and activation of the ZSCAN4 transcriptional program. These changes to the chromatin landscape, which lead cells to acquire a 2C-like identity, are accompanied by the transcriptional up-regulation of active LINE-1 elements and impede differentiation potential. These data provide previously unknown insights into the epigenetic regulation of early development. Moreover, this work identifies satellite DNA arrays as potential cis-regulatory platforms, which, together with their cognate KZFPs, may regulate developmental fate transitions, through barcoding and switching off master transcription factor genes.

By analogy, we predict that similar mechanisms would be operational in human systems but are likely to occur through human-specific satellite DNAs and KZFPs. ZSCAN4 governs chromatin remodeling in human cancer stem cells (*44*). Further pursuing these pathways in human cells may, therefore, also provide new insight into how cancers remodel their chromatin when epigenetic silencing mechanisms break down (*43*). Last, these results pinpoint active LINE-1 elements as a hallmark of genome dysregulation, linked to loss of heterochromatin roadblocks and which may be a common feature of a range of autoimmune conditions and cancers (*40*).

## MATERIALS AND METHODS

### Experimental design

This project was designed with the aim to target ZFP819 for depletion using shRNAs to mimic its natural lower expression at the 2C stage of development to ascertain whether this could induce a cell fate switch. Using an shRNA approach enabled us to determine the reproducibility of the phenotype in a range of ESC lines from different strains of mice. This was appropriate because ZFP819 and the satellite repeat ZP3AR that it binds to are more than 12 million years old and represent a gene regulatory pathway conserved in rodents.

### Cell culture and reagents

Mouse embryonic stem cell lines (ES3, referred to as mESCs, J1, E14, and MERVL*-tdTomato* reporter E14 mESCs, the latter of which were a gift from W. Reik’s laboratory) were cultured in Glasgow minimum essential medium (GMEM) (Sigma-Aldrich) supplemented with penicillin/streptomycin (100 U/ml) (Gibco, Thermo Fisher Scientific), 10% heat-inactivated ESC-tested fetal calf serum (FCS) (Life Technologies), 2 mM l-glutamine (Gibco, Thermo Fisher Scientific), 1 mM sodium pyruvate (Sigma-Aldrich), 1× minimum essential medium (MEM) nonessential amino acids (Gibco, Thermo Fisher Scientific), 0.1 mM 2-mercaptoethanol (Life Technologies), and leukemia inhibitory factor (LIF) (1000 U/ml; Chemicon) and grown at 5% CO_2_ at 37°C. mESC lines were split 1:4 every 2 days using accutase or trypsin. Human embryonic kidney (HEK) 293T cells were cultured in Dulbecco’s minimum essential medium (DMEM) supplemented with penicillin/streptomycin (100 U/ml) and 10% heat-inactivated FCS and grown at 5% CO_2_ at 37°C and split 1:5 every 2 days using trypsin. ShRNA vector plasmids (pLKO.1) were ordered from Dharmacon (see the Supplementary Materials for sequences). GFP was cloned in place of the puromycin resistance cassette, and GFP versions were used for GFP sorting experiments. Vesicular stomatitis virus G protein (VSV-G)–pseudotyped lentiviral vectors were produced by cotransfecting HEK293T cells in 10-cm plates with 1.5 μg of the shRNA plasmid, 1 μg of p8.91, and 1 μg of pMDG2-encoding VSV-G. Media was changed 1 day after transfection, and supernatant was harvested 48 hours after transfection and concentrated via ultracentrifugation (20,000*g* for 2 hours at 4°C). Puromycin selection was performed 2 days after transduction overnight (and control cells verified to die) and samples were harvested 5 days after selection.

### Western blotting

Cells were washed once in ice-cold phosphate-buffered saline (PBS) and lysed using prechilled homemade radioimmunoprecipitation assay buffer [150 mM NaCl, 1% Triton X-100, 0.5% sodium deoxycholate, 0.1% SDS, 50 mM tris (pH 8.0), and protease inhibitor cocktail (cOmplete, Mini, EDTA-free, Roche)] for 30 min at 4°C. Cell lysates were cleared of debris by centrifugation (12,000*g*, 20 min, 4°C). Protein quantification assay (BCA Protein Assay Kit, Millipore) was used to standardize lysate samples for loading. Samples were mixed with NuPAGE lithium dodecyl sulfate (LDS) sample buffer (Thermo Fisher Scientific), heated at 95°C for 5 min, and then resolved on either precast (Bio-Rad) or handcast 10% SDS–polyacrylamide gels in tris/glycine/SDS buffer in Mini-Protean tanks (Bio-Rad), followed by wet transfers onto polyvinylidene difluoride or nitrocellulose membranes, blocked in 5% nonfat dried milk in Tris Buffered Saline-Tween (TBS-T) [TBS, 0.1% Tween-20 (Sigma-Aldrich)] and incubated with relevant primary antibodies: anti–proliferating cell nuclear antigen, anti-ZSCAN4, anti-POU5F1, anti-HA, anti-TRIM28, anti-SETDB1, anti-H3, and anti-H3K9me3. See the Supplementary Materials for antibody details. Secondary antibodies were horseradish peroxidase–conjugated (GE Healthcare) or IRDye 650 dye–conjugated (LI-COR). Membranes were developed using enhanced chemiluminescence (ECL) kits (ECL, Prime or Select kits from Amersham) or a LI-COR Odyssey Imager.

### NPC differentiation

SOX1-GFP reporter mESCs [46C ESCs (*45*)] were a gift from the A. Smith laboratory and were cultured as previously described (*46*) in N2B27 media: DMEM/F12 (Gibco, Thermo Fisher Scientific), Neurobasal (Gibco, Thermo Fisher Scientific), N2 (Gibco, Thermo Fisher Scientific), and B27 (Gibco, Thermo Fisher Scientific) and supplemented with 0.08% BSA and penicillin/streptomycin (100 U/ml), under 2i/LIF culture conditions with LIF (1000 U/ml), 1 μM PD0325901, and 3 μM CHIR99021. ESCs were transduced with shRNA vectors, and neural differentiation was initiated on day 3 after puromycin selection and was carried out according to Gouti *et al.* (*47*) with some modifications: Briefly, mESCs were collected and plated onto laminin-coated six-well plates at a density of 65,000 cells per well in N2B27 media [as above but with N2 Supplement-B from STEMCELL Technologies and laminin (1 μg/ml)] supplemented with basic fibroblast growth factor (bFGF) (10 ng/μl; R&D Systems) for days 1 to 2 of neural differentiation and then in N2B27 without bFGF for days 3 to 5 at 7% CO_2_. On day 5, cells were collected and analyzed by flow cytometry (ACEA NovoCyte 3000) to measure GFP expression.

### Flow cytometry and cell sorting

Cells were trypsinized and harvested in media, washed with cold PBS, resuspended in flow cytometry buffer (PBS 2% FBS and 5 mM HEPES), and run on a BD FACSCalibur acquiring 10,000 events per sample to meet statistical robustness. Data were analyzed using FlowJo (Tree Star version 10.3.0). The flow cytometry gating strategy involved gating on live cells and then using a negative control sample to set a gate of GFP or tdTomato-positive cells. For cell sorting, cells were resuspended in flow cytometry buffer and sorted using a BD FACSAria cell sorter. The gating strategy involved gating on live cells, then on single cells, and, finally, on GFP-positive cells. Cells were sorted into GFP-negative, GFP^Dim^, and GFP^Bright^ fractions.

### Coimmunoprecipitation

Full-length (long) and a naturally occurring truncated (short) isoform of ZFP819 were amplified from mESC complementary DNA (cDNA) using a primer set designed on the canonical isoform and cloned into a pRRLSIN.cPPT.PGK-GFP.WPRE lentiviral vector in place of GFP. Versions with and without a triple HA tag were constructed. HA-tagged constructs were used for coimmunoprecipitation: HEK293T cells were transfected with the ZFP819-expressing plasmids or ZFP809 or the GFP-expressing plasmid as controls, using FuGENE6 (Promega). Cells were lysed day 2 after transfection with a 0.1% NP-40 lysis buffer [50 mM tris-HCl (pH7.5), 150 mM NaCl, 0.1% Nonidet P40, and glycerol 10%] in the presence of protease inhibitors (cOmplete, Mini, EDTA-free, Roche) on ice for 30 min. Protein content was measured using a bicinchoninic acid (BCA) assay according to the manufacturer’s instructions (Pierce BCA Protein Assay Kit, Thermo Fisher Scientific). Ten percent of the lysate was removed as a control (pre-IP), and the remaining lysates were immunoprecipitated on Pierce anti-HA magnetic beads at 4°C overnight, after which 10% of the supernatant was removed (post-IP). The beads were washed with ice-cold lysis buffer and resuspended in loading buffer with reducing agent (IP). All samples (pre-IP, post-IP, and IP) were boiled at 95°C for 5 min and resolved on 10% SDS–polyacrylamide gels, and Western blots were carried out to detect interactions.

### RNA quantification

Total RNA was extracted using RNeasy Micro kit columns (QIAGEN) and DNase-treated according to the manufacturer’s instructions (Ambion AM1907). RNA (500 ng) was reverse-transcribed using random primers and SuperScript II Reverse Transcriptase (Thermo Fisher Scientific). Control reactions were always performed in the absence of reverse transcriptase and used for RT-qPCR in parallel to cDNA to verify that there was no preexisting DNA contamination. cDNA was diluted in nuclease-free water, and gene expression levels were quantified using RT-qPCR using the QuantStudio 5 Real-Time PCR System (Applied Biosystems) or the LightCycler 480 System (Roche). SYBR Green Fast PCR Mastermix (Life Technologies) was used. Cycle threshold (CT) values for the test genes were normalized against those of *Gapdh* or *Cox6a1* using the –ΔΔCt method to calculate fold change. See the Supplementary Materials for primer sequences.

### Total RNA-seq and analyses

RNA was quality-checked on a 4200 Tapestation using the RNA ScreenTape assay (Agilent Technologies, Wokingham, UK), and the RNA concentration was measured using a Qubit RNA Broad Range kit (Life Technologies, Paisley, UK). Total RNA samples were processed using KAPA’s stranded RNA HyperPrep RiboErase kit using an input of 500 ng per sample. Samples were sequenced on a NextSeq 500 instrument (Illumina Cambridge, Chesterford, UK) after pooling libraries in equimolar quantities, using a 2× 151–base pair (bp) paired-end run, resulting in more than 15 million reads per sample. Illumina’ s bcl2fastq Conversion Software was used to demultiplex data and generate fastq files. TrimGalore v0.4.1 (*48*) was used to trim for quality and remove adaptors, and fastq files were checked for quality before and after trimming with FastQC v0.11.8 (*49*). Trimmed reads were aligned to the mouse genome (v GRCm38), which was downloaded from the Illumina iGenomes portal (http://support.illumina.com/sequencing/sequencing_software/igenome.ilmn) using Tophat 2.1.0 (*50*) with the *--*max-multihits 100 recommended by TEtranscripts (*51*). The number of read counts per gene and repeat family was calculated using the multi option from TEcount 2.0.3 with the repeat annotation provided for GRCm38 and the gene annotation from iGenomes. The BioConductor package DESeq2 (*52*), under R v4.0.3, was used to perform differential expression analysis using the TEcounts output, where the Approximate Posterior Estimation method (*53*) was used to shrink the logarithmic fold change. *P* values were adjusted for multiple testing with the Benjamini-Hochberg false discovery rate procedure, which is built into the DESeq2 default analysis pipeline. Genes were considered as significantly differentially expressed when the adjusted *P* values were <0.05. Gene set enrichment analysis was performed with the Bioconductor package fgsea v1.8.0 using abs(log_2_fold change) × [−log_10_(*P* value)] to rank genes. Because the genome version used did not contain the sequence corresponding to *Dux*, Bowtie2 v.2.2.5 (*54*) was used to align directly to the *dux* repeat as has been done previously (*24*). Expression coverage tracks were generated from an alignment to GRCm38 using STAR (*55*) to output one random location for multimapping reads by applying the following parameters: *--*outFilterMultimapNmax 5000 --outSAMmultNmax 1 --outFilterMismatchNmax 999 --outFilterMismatchNoverLmax 0.06. The alignment files were used to obtain coverage scaled by library size through the genomecov tool from bedtools (v2.28.0). The resulting bedGraph files were converted to BigWigs using the UCSC Genome Browser utilities, and the BigWig tracks were visualized using the Integrative Genomics Viewer or Python 3.9.9 using pybigwig 0.3.18 and matplotlib libraries. RNA-seq data from GSE66582 were downloaded using the Sequence Read Archive (SRA) toolkit, and reads were mapped to the GRCm38 version of the mouse genome using STAR as above. The number of read counts per gene was calculated using HTSeq-Count (*56*). Count files were used to calculate transcripts per million (TPM) using R.

### Chromatin immunoprecipitation

ES3 mESCs were transduced with ZFP819 and ZFP809-expressing lentiviral vectors (pRRLSIN.cPPT.PGK-ZFP.WPRE vectors with a triple HA tag). Protein expression of ZFP819Long-HA and ZFP809-HA was verified using Western blotting. Samples were washed twice (in PBS + 2% FCS), counted to normalize by cell number, cross-linked (10-min rotation in 1% formaldehyde), quenched with glycine (at 125 mM on ice), washed three times (PBS), and snap-frozen at 10^7^ cells per tube. Cells were resuspended in sequential ChIP lysis buffers and then in 900 μl of sonication buffer on ice (10mM tris at pH 8, 200 mM NaCl, 1 mM EDTA, 0.5 mM EGTA, 0.1% NaDOC, 0.25% NLS, and protease inhibitors). Where a Covaris sonicator was used, 900 μl was sonicated in one Covaris glass tube with the following settings: 20% duty cycle, intensity 5, 200 cycles per burst, 30 min. For sonication with a Bioruptor Pico, 300-μl aliquots per 1.5-ml tube were sonicated (*57*). IPs were performed in duplicate as previously described (*58*) using an HA antibody (Ab) (BioLegend), and DNA was extracted (QIAGEN MinElute PCR purification kit, catalog no. 28004) for PCR and sequencing. See the Supplementary Materials for primers.

### ChIP-seq analyses

Single-end reads were mapped to the mouse genome mm10 using Bowtie2 (*54*). For multimapping reads, one random alignment was reported. BigWig files, heatmaps, and profile plots were generated using deepTools (v3.4.2) (*59*). Genome track views of BigWig files were created in the Integrative Genomics Viewer (IGV) browser (v2.8.10). ChIP-sequencing (ChIP-seq) enriched peaks were called by MACS2 (v2.1.2), and the common peaks were merged using bedtools and used for subsequent analysis. The circos plots were constructed with the circlize R package (*60*). Plots represent genomic density tracks over overlapping genomic windows. ZP3AR copies bound and unbound by ZFP819 were identified using bedtools using the ZFP819 peak calls. Sequences corresponding to the classified ZP3ARs were retrieved using the getfasta command from bedtools. Mafft 7.480 was used to perform the multiple sequence alignments, which were used as input for hmmbuild from HMMER 3.1b2 to build hidden Markov models. Hmmemit was used to calculate the majority consensus for bound and unbound ZP3AR sequences. ZP3AR % divergence to consensus was obtained from the RepeatMasker output file for mm10. A two-sided unpaired *t* test was used to assess significance. Bruce4 mESC ENCODE peaks of p300, H3K27ac, and H3K9me3 were downloaded from the ENCODE data portal. Liftover was used to convert the coordinates to version GRCm38 of the mouse genome. To assess the significance of overlap between ZFP819 peaks and each of the peak files downloaded from ENCODE, the number of overlaps between datasets and randomized coordinates was recorded and used to calculate a *P* value corresponding to (1+ number of randomizations with a number of overlaps greater than the observed value)/1000 randomizations. Observed versus expected ratios were calculated on the basis of the proportion of the genome covered by the corresponding ENCODE ChIP peaks and the total number of ZFP819 peaks. ChIP-seq reads from GSE94323 were retrieved using the SRA toolkit and aligned using bowtie2 v2.2.5. The bdgcmp command of MACS2 was used to generate fold enrichment over input tracks from the bedGraph pileup generated by the −B option of the callpeak MACS2 command. The same randomization approach described above was used to assess significance.

### CUT&RUN

We followed the EpiCypher CUTANA CUT&RUN Protocol (v1.6) (https://epicypher.com/resources/protocols/cutana-cut-and-run-protocol/). Briefly, CUT&RUN was performed using 100,000 sorted GFP^Bright^ cells (per antibody/sample combination). Cells were washed twice [20 mM HEPES (pH 7.5), 150 mM NaCl, 0.5 mM spermidine, and 1× protease inhibitors (cOmplete, Mini, EDTA-free, Roche)], attached to Concanavalin A–coated magnetic beads (BioMagPlus, Generon), and preactivated in activation buffer [20 mM HEPES (pH 7.9), 10 mM KCl, 1 mM CaCl_2_, and 1 mM MnCl_2_]. The sample and bead slurry was resuspended in 50 μl of antibody buffer [20 mM HEPES (pH 7.5), 150 mM NaCl, 0.5 mM spermidine, 1× protease inhibitors, 0.01% (w/v) digitonin, and 2 mM EDTA] containing primary antibody. One microgram of anti-H3K9me3, anti-H3K27ac, or immunoglobulin G (IgG) control Ab was incubated overnight at 4°C with gentle shaking. The sample slurry was washed 3× in cold digitonin buffer [20 mM HEPES (pH 7.5), 150 mM NaCl, 0.5 mM spermidine, 1× Roche cOmplete protease inhibitors, and 0.01% digitonin]. Protein A and protein G fused to micrococcal nuclease (pAG-MNase) (2.5 μl per tube; CUTANA pAG-MNase, Epicypher) was added to 50 μl of digitonin buffer and incubated with the bead-cell slurry at room temperature for 10 min. The beads were washed twice and resuspended in 50 μl of cold digitonin buffer. Cleavage by the pAG-MNase was activated by the addition of 2 mM CaCl_2_ (final) and incubating at 4°C for 2 hours. The reaction was quenched by the addition of 33 μl of Stop buffer [340 mM NaCl, 20 mM EDTA, 4 mM EGTA, glycogen (50 μg/ml), and ribonuclease A (50 μg/ml)], vortexing, and incubating for 10 min at 37°C to release genomic fragments. A magnetic rack was used to separate cells and beads from the supernatant, which was purified with the MinElute PCR Purification Kit (QIAGEN). Illumina sequencing libraries were prepared using the NEBNext Ultra II DNA Library Prep Kit for Illumina and NEBNext Multiplex Oligos for Illumina (New England BioLabs), pooled in equimolar quantities, and sequenced on NovaSeq6000 for 150 paired-end reads.

### CUT&RUN analyses

Sequencing reads were quality-trimmed, and adaptors were removed using TrimGalore v0.4.1. FastQC v0.11.8 was used before and after trimming to check for quality. Trimmed reads were aligned with STAR (*55*) to the mouse genome v GRCm38 as described above, with the parameters necessary to obtain one random location for multimapping reads. BedGraph files of genomic coverage scaled by library size were generated with the genomecov tool from bedtools and converted to BigWig files. BigWig files were read into Python3, and the coverage in the indicated genomic positions was retrieved in 100-bp windows using the pybigwig library. The resulting values were represented as heatmaps or profile plots. In the case of profile plots, the trimmed mean (excluding values corresponding to the top and bottom 0.5 percentile) was calculated across corresponding 100-bp bins in the regions surrounding and including the indicated features, where features were scaled to the same number of bins. Boxplots were generated using the mean signal across all bins corresponding to each genomic feature. H3K27ac peaks were called from the STAR alignments described above, where IgG replicates were pooled together and used as control to call peaks using epic2 with a false discovery rate cutoff of 0.001. Peaks called for each CUT&RUN replicate independently were used as input for the Bioconductor package DiffBind, where an occupancy-based analysis was performed using the dba.overlap function. Subsets of peaks occurring in the knockdown (KD) replicates alone or in KD and empty were overlapped to repeat annotations using pybedtools to obtain the fractions, out of the total length per peak subset, which corresponded to repeats classified at either the class or family/superfamily level.

### Hi-C data analysis

Pairs of pooled replicates were downloaded from Gene Expression Omnibus, accession GSE82185 (*61*). Files were parsed to accommodate input specifications of juicer (https://doi.org/10.1016/j.cels.2016.07.002). Hi-C files were created with the pre command of juicer tools on chromosome 7. The resulting file was loaded into juicebox to enable visualization of data. Compartments were called by running the eigenvector command from juicer tools, using the Knight-Ruiz balancing normalization at a 500-kb resolution. Eigenvector values were assigned to 500-kb windows and further parsed to generate a bedpe file, which could be loaded into juicebox.

### Statistical analyses

All data are presented with error bars showing SD, and statistical significance was assessed using two-tailed, paired Student’s *t* tests, or other statistical tests where stated (see figure legends for details) using GraphPad Prism. The number of biological replicates is stated in the figure legends. For flow cytometry, 10,000 events were recorded. A *P* value of <0.05 was considered statistically significant (****P < 0.0001, ***P < 0.001, **P < 0.01, and *P < 0.05). *P* values are stated in the legends.

## Notes

View/request a protocol for this paper from *Bio-protocol*.
