# Supplementary material for: A satellite DNA array barcodes chromosome 7 and regulates totipotency via ZFP819
Source: Sci Adv. 2022 Oct 28;8(43):eabp8085. doi: 10.1126/sciadv.abp8085 (PMC9616502; doi:10.1126/sciadv.abp8085)
Supplement: Supplementary file 1 — Figs. S1 to S9 Tables S1 and S2 [file sciadv.abp8085_sm.pdf]

Science Advances
AAAS

Supplementary Materials for

A satellite DNA array barcodes chromosome 7 and regulates totipotency via ZFP819

Liane P. Fernandes *et al.*

Corresponding author: Helen M. Rowe, h.rowe@qmul.ac.uk

Sci. Adv. **8**, eabp8085 (2022)
DOI: 10.1126/sciadv.abp8085

The PDF file includes:

Figs. S1 to S9
Tables S1 and S2
Legend for data file S1

Other Supplementary Material for this manuscript includes the following:

Data file S1

Fig. S1

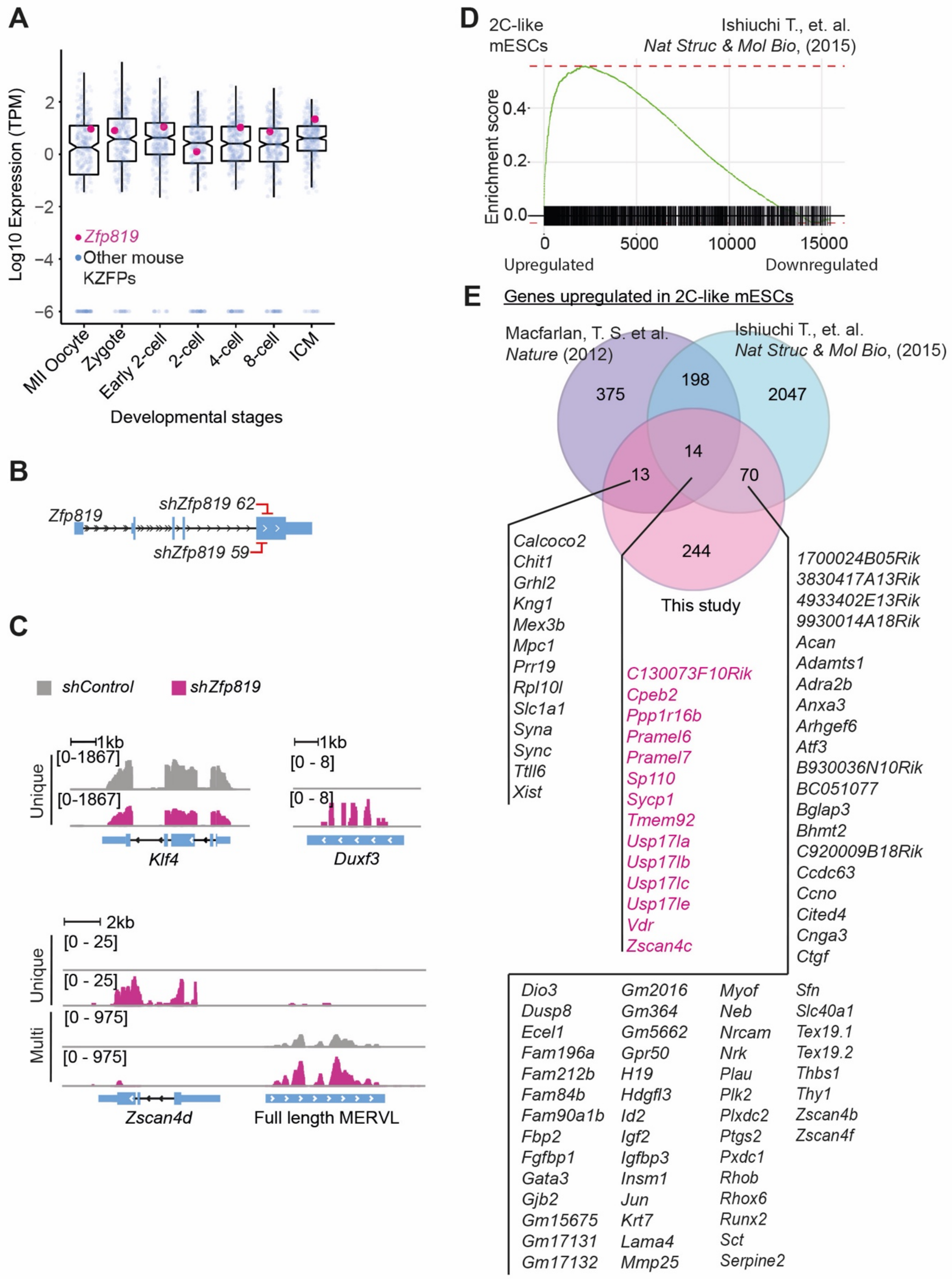
A
Log10 Expression (TPM)
2
0
−2
−4
−6
Zfp819
Other mouse KZFPs
MII Oocyte
Zygote
Early 2-cell
2-cell
4-cell
8-cell
ICM
Developmental stages
B
Zfp819
shZfp819 62
shZfp819 59
C
shControl
shZfp819
1kb
[0-1867]
Unique
[0-1867]
Klf4
1kb
[0 - 8]
[0 - 8]
Duxf3
2kb
[0 - 25]
Unique
[0 - 25]
[0 - 975]
Multi
[0 - 975]
Zscan4d
Full length MERVL
D
2C-like mESCs
Ishiuchi T., et. al.
Nat Struc & Mol Bio, (2015)
Enrichment score
0.4
0.2
0.0
0
5000
10000
15000
Upregulated
Downregulated
E
Genes upregulated in 2C-like mESCs
Macfarlan, T. S. et al.
Nature (2012)
Ishiuchi T., et. al.
Nat Struc & Mol Bio, (2015)
375
198
2047
14
13
70
244
This study
Calcoco2
Chit1
Grhl2
Kng1
Mex3b
Mpc1
Prr19
Rpl10l
Slc1a1
Syna
Sync
Ttll6
Xist
C130073F10Rik
Cpeb2
Ppp1r16b
Pramel6
Pramel7
Sp110
Sycp1
Tmem92
Usp17la
Usp17lb
Usp17lc
Usp17le
Vdr
Zscan4c
1700024B05Rik
3830417A13Rik
4933402E13Rik
9930014A18Rik
Acan
Adamts1
Adra2b
Anxa3
Arhgef6
Atf3
B930036N10Rik
BC051077
Bglap3
Bhmt2
C920009B18Rik
Ccdc63
Ccno
Cited4
Cnga3
Ctgf
Dio3
Dusp8
Ecel1
Fam196a
Fam212b
Fam84b
Fam90a1b
Fbp2
Fgfbp1
Gata3
Gjb2
Gm15675
Gm17131
Gm17132
Gm2016
Gm364
Gm5662
Gpr50
H19
Hdgfl3
Id2
Igf2
Igfbp3
Insm1
Jun
Krt7
Lama4
Mmp25
Myof
Neb
Nrcam
Nrk
Plau
Plk2
Plxdc2
Ptgs2
Pxdc1
Rhob
Rhox6
Runx2
Sct
Serpine2
Sfn
Slc40a1
Tex19.1
Tex19.2
Thbs1
Thy1
Zscan4b
Zscan4f

Fig. S1. mESCs transition to a 2C-like state upon loss of ZFP819.

(A) Transcript levels of 327 KZFPs, including *Zfp819 mRNA* (marked in pink), through early developmental stages. TPM, transcripts per million. ICM, inner cell mass. (**B)** Location of sites targeted by shRNAs within *Zfp819*. Note that another shRNA against *Zfp819* called *shZfp819 58* targeted against the 3'UTR was the least effective shRNA and therefore was not pursued but it appears in the Western data in Fig. 1H. (**C)** Genome track views of RNA-seq data showing BigWig tracks to demonstrate expression changes at example genes (unique mapping) and TEs (multi-mapping) in *Zfp819*-depleted mESCs. Coverage normalized by reads per million. (**D)** Gene set enrichment analysis (GSEA) profile illustrates upregulation of 2-cell (2C)-like genes upon ZFP819-depletion. 2C genes were acquired from (*27*). *P*-adjusted value = 0.0003 (**E)** Venn-diagram of common upregulated 2C genes between this study and two independent studies (*3, 27*).

Fig. S2

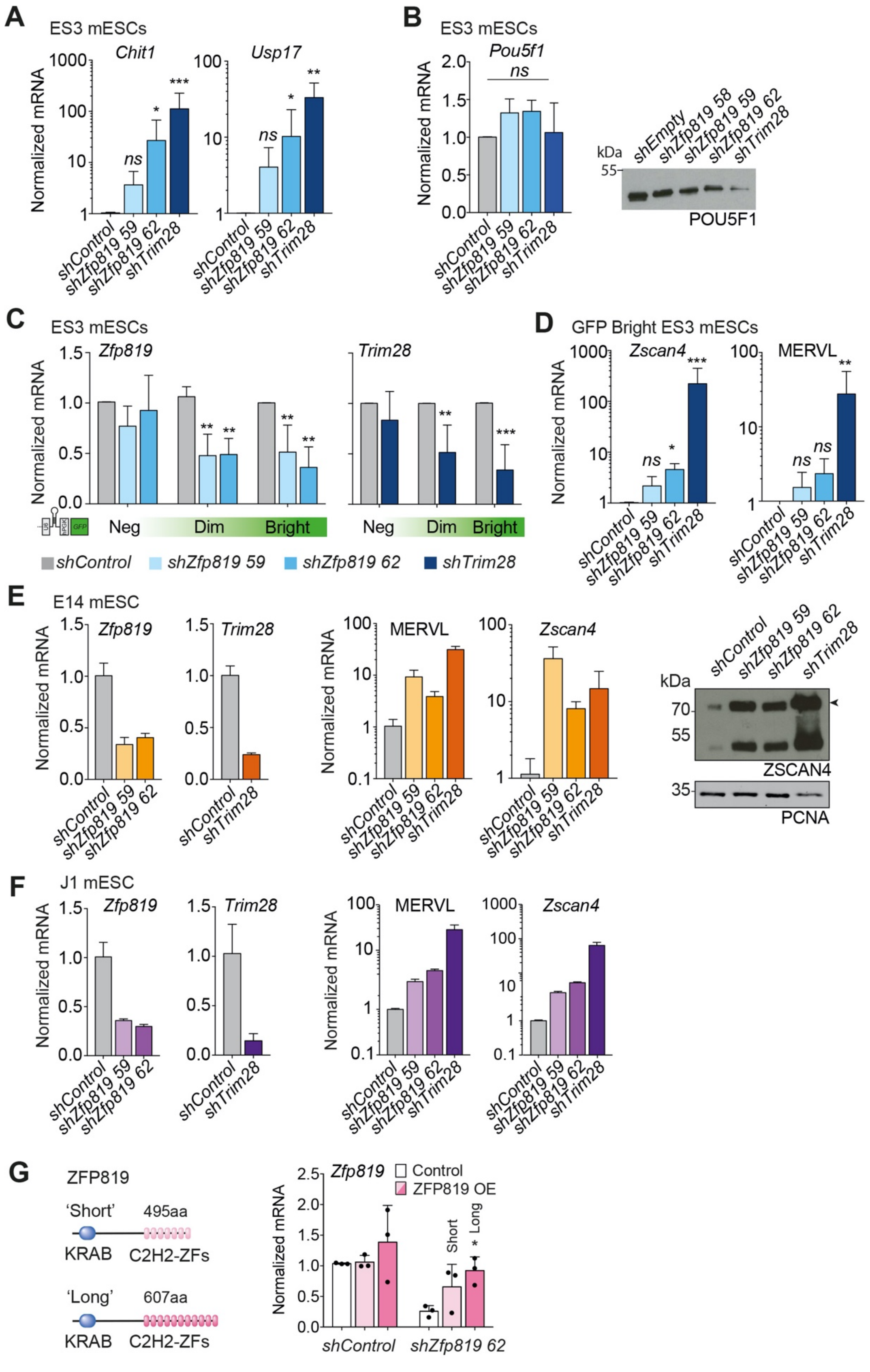
A
ES3 mESCs
Chit1
Usp17
Normalized mRNA
ns
*
**
***
shControl
shZfp819 59
shZfp819 62
shTrim28
B
ES3 mESCs
Pou5f1
ns
kDa
55
shEmpty
shZfp819 58
shZfp819 59
shZfp819 62
shTrim28
POU5F1
C
ES3 mESCs
Zfp819
Trim28
Neg
Dim
Bright
shControl
shZfp819 59
shZfp819 62
shTrim28
D
GFP Bright ES3 mESCs
Zscan4
MERVL
E
E14 mESC
Zfp819
Trim28
MERVL
Zscan4
kDa
70
55
35
ZSCAN4
PCNA
F
J1 mESC
Zfp819
Trim28
MERVL
Zscan4
G
ZFP819
'Short'
495aa
KRAB
C2H2-ZFs
'Long'
607aa
Zfp819
Control
ZFP819 OE
Short
Long
shControl
shZfp819 62

Fig. S2. Loss of ZFP819 governs a 2C switch in different mESC models.
(A) RT-qPCR validation of 2C genes upregulated in *shControl*, *shZfp819* and *shTrim28* mESCs 5 days post puromycin selection. *Chit1*: *P*-value = 0.0143 (*shZfp819 62)* and *P*- value = 0.0008 (*shTrim28); Usp17*: *P*-value = 0.0269 (*shZfp819 62)* and *P*-value = 0.0011 (*shTrim28)* (two-tailed Student's *t* test, compared to *shControl)***.** **(B)** RT-qPCR (Left) and Western blot analysis (Right) of *Pou5f1* mRNA and POU5F1/OCT4 protein levels, respectively, in *shControl*, *shZfp819* and *shTrim28* mESCs. Data show mean ± s.d of 3 independent experiments (two-tailed Student's *t* test, compared to *shControl*). Ns: not significant. (**C)** RT-qPCR analysis of *Zfp819* and *Trim28* gene expression in ZFP819-depleted (Left) and TRIM28-depleted mESCs (Right), respectively, following selection of knockdown cells by GFP sorting and comparing the GFP^{bright} to the GFP^{dim} fraction. Data show mean ± s.d of 3 independent experiments. GFP^{dim}: *P*-value = 0.0012 (*shZfp819 59), P*-value = 0.0015 (*shZfp819 62)* and *P*-value = 0.0060 (*shTrim28);* GFP^{bright}: *P*-value = 0.0068 (*shZfp819 59), P*-value = 0.0004 (*shZfp819 62)* and *P*-value = 0.0003 (*shTrim28)* (two-tailed Student's *t* test, compared to *shControl*). (**D)** RT-qPCR detection of 2C transcripts in ZFP819- and TRIM28- depleted GFP^{bright} mESCs. TRIM28 depletion was included as a positive control. Data show mean ± s.d of 3 technical replicates. *Zscan4*: *P*-value = 0.0176 (*shZfp819 62)* and *P*-value = 0.0005 (*shTrim28);* MERVL*:* *P*-value = 0.0011 (*shTrim28)* (two-tailed Student's *t* test, compared to *shControl*). (**E) and (F)** Recapitulation of the 2C-switch phenotype in E14 (129/Ola strain) and J1 (129S4/SvJae) mESC lines. Western blot analysis shows ZSCAN4 expression in *shZfp819* and *shTrim28* E14 cells. (**G)** Domains of ZFP819 Short and Long isoforms (Left). RT-qPCR validation of ZFP819 isoform overexpression (Right). Data are mean ± s.d. of 3 independent experiments. *P*-values: Short isoform = 0.1625; long isoform = 0.0241 (two-tailed paired Student's *t* test, compared to *shControl*).

Fig. S3

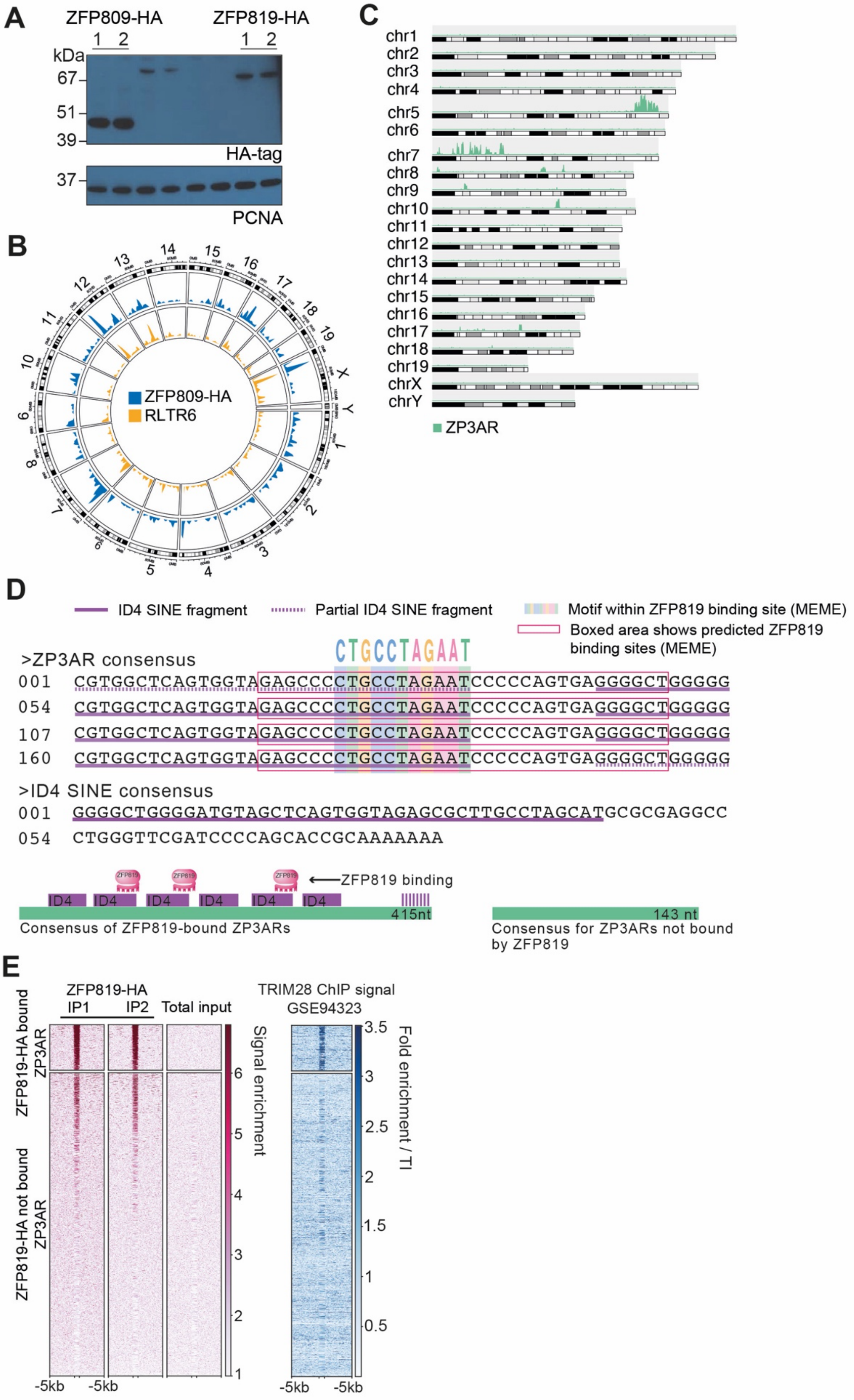
A
ZFP809-HA
ZFP819-HA
1 2
1 2
kDa
67
51
39
HA-tag
37
PCNA
B
ZFP809-HA
RLTR6
C
chr1
chr2
chr3
chr4
chr5
chr6
chr7
chr8
chr9
chr10
chr11
chr12
chr13
chr14
chr15
chr16
chr17
chr18
chr19
chrX
chrY
ZP3AR
D
ID4 SINE fragment
Partial ID4 SINE fragment
Motif within ZFP819 binding site (MEME)
Boxed area shows predicted ZFP819 binding sites (MEME)
CTGCCTAGAAT
>ZP3AR consensus
001 CGTGGCTCAGTGGTAGAGCCCCTGCCTAGAATCCCCCAGTGAGGGGCTGGGGG
054 CGTGGCTCAGTGGTAGAGCCCCTGCCTAGAATCCCCCAGTGAGGGGCTGGGGG
107 CGTGGCTCAGTGGTAGAGCCCCTGCCTAGAATCCCCCAGTGAGGGGCTGGGGG
160 CGTGGCTCAGTGGTAGAGCCCCTGCCTAGAATCCCCCAGTGAGGGGCTGGGGG
>ID4 SINE consensus
001 GGGGCTGGGGATGTAGCTCAGTGGTAGAGCGCTTGCCTAGCATGCGCGAGGCC
054 CTGGGTTCGATCCCCAGCACCGCAAAAAAA
ZFP819 binding
ID4
415nt
Consensus of ZFP819-bound ZP3ARs
143 nt
Consensus for ZP3ARs not bound by ZFP819
E
ZFP819-HA
IP1
IP2
Total input
TRIM28 ChIP signal
GSE94323
ZFP819-HA bound ZP3AR
ZFP819-HA not bound ZP3AR
Signal enrichment
Fold enrichment / TI
-5kb
-5kb

Fig. S3. ZFP819 targets a satellite repeat, ZP3AR coating chromosomes 5 and 7.
(A) Western blot analysis of ZFP819-HA and ZFP809-HA overexpression in mESCs. HA: hemagglutinin. The HA tag is present three times to improve immunoprecipitation. **(B)** ZFP809-HA ChIP peaks and RLTR6 repeat instance densities illustrated on a genome-wide visualization circos plot. **(C)** Genome-wide distribution of ZP3AR represented on a karyotype plot to show more detail than the circos plot in Fig. 2a **(D)** Consensus sequences of ZP3AR and ID4 SINE from the *Dfam* database. The ZFP819-HA predicted binding site (the sequence under the ChIP-seq peaks) was determined using MEME (https://meme-suite.org/meme/) and is boxed (pink box) with the high confidence core motif (MEME) within it highlighted (multicolour) and ID4 SINE fragments are underlined in purple (solid and dotted) (top). The ZFP819 binding site occurs four times in the consensus ZP3AR sequence. The consensus sequences of ZFP819-HA-bound vs. not bound ZP3ARs are given (bottom). ZFP819-HA binding and ID4 SINE fragments are annotated. The ZFP819-HA-bound and not bound ZP3AR consensus sequences were generated using HMMER. SINE: Short INterspersed Element. **(E)** Heatmap of ZFP819-HA ChIP normalized read coverage (Left) and TRIM28 ChIP signal normalized to total input (Right) at ZP3ARs sorted by ZFP819-HA binding intensity. ZP3AR elements were separated into ZFP819-HA-bound and not bound as determined by the presence of high-confidence ZFP819-HA ChIP peaks from Fig. 2C.

Fig. S4

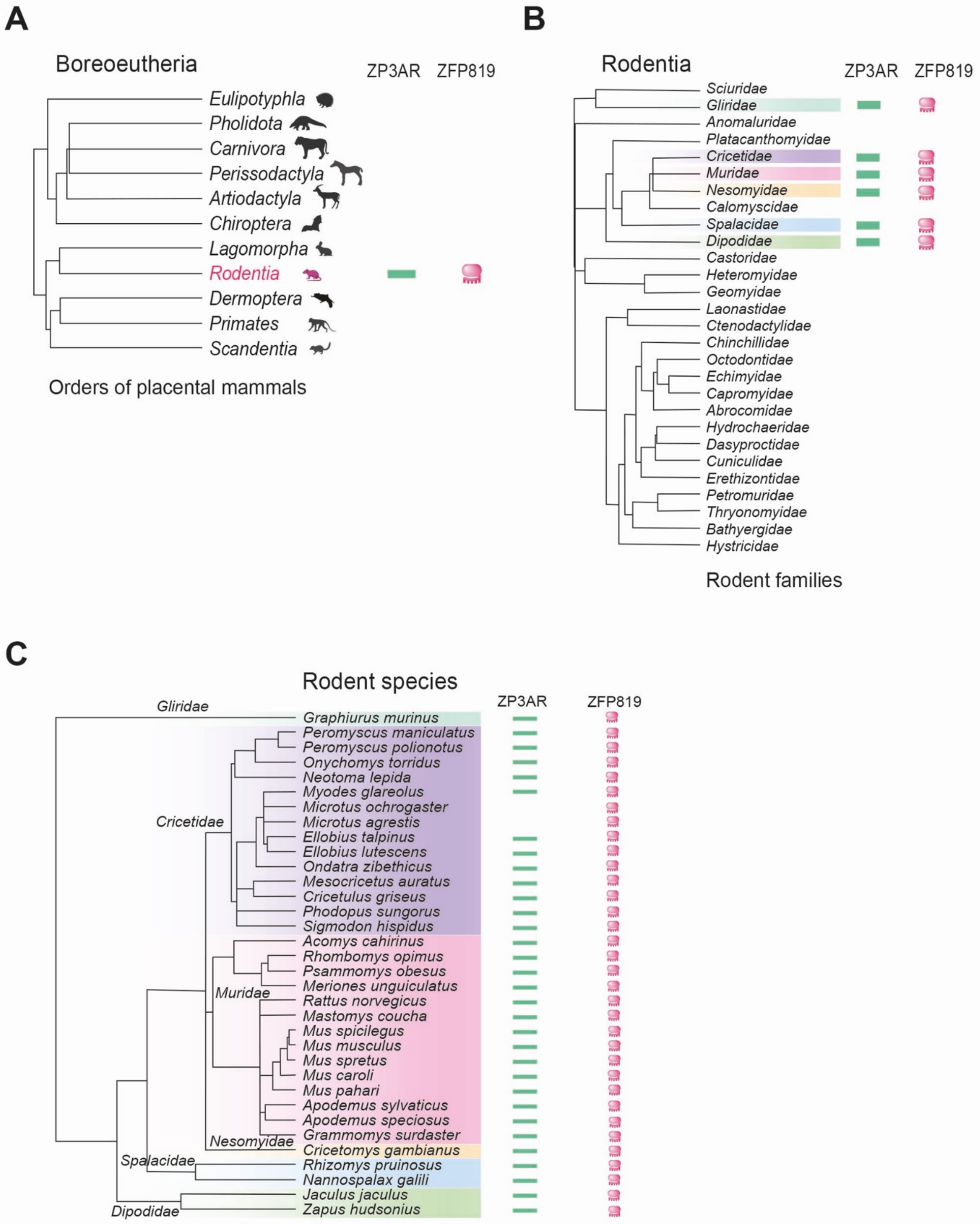
A
Boreoeutheria
ZP3AR
ZFP819
Eulipotyphla
Pholidota
Carnivora
Perissodactyla
Artiodactyla
Chiroptera
Lagomorpha
Rodentia
Dermoptera
Primates
Scandentia
Orders of placental mammals
B
Rodentia
ZP3AR
ZFP819
Sciuridae
Gliridae
Anomaluridae
Platacanthomyidae
Cricetidae
Muridae
Nesomyidae
Calomyscidae
Spalacidae
Dipodidae
Castoridae
Heteromyidae
Geomyidae
Laonastidae
Ctenodactylidae
Chinchillidae
Octodontidae
Echimyidae
Capromyidae
Abrocomidae
Hydrochaeridae
Dasyproctidae
Cuniculidae
Erethizontidae
Petromuridae
Thryonomyidae
Bathyergidae
Hystricidae
Rodent families
C
Rodent species
ZP3AR
ZFP819
Gliridae
Cricetidae
Muridae
Nesomyidae
Spalacidae
Dipodidae
Graphiurus murinus
Peromyscus maniculatus
Peromyscus polionotus
Onychomys torridus
Neotoma lepida
Myodes glareolus
Microtus ochrogaster
Microtus agrestis
Ellobius talpinus
Ellobius lutescens
Ondatra zibethicus
Mesocricetus auratus
Cricetulus griseus
Phodopus sungorus
Sigmodon hispidus
Acomys cahirinus
Rhombomys opimus
Psammomys obesus
Meriones unguiculatus
Rattus norvegicus
Mastomys coucha
Mus spicilegus
Mus musculus
Mus spretus
Mus caroli
Mus pahari
Apodemus sylvaticus
Apodemus speciosus
Grammomys surdaster
Cricetomys gambianus
Rhizomys pruinosus
Nannospalax galili
Jaculus jaculus
Zapus hudsonius

Fig. S4. Conservation of *Zfp819* and ZP3AR.

(A) Phylogenetic tree of Boreoeutherian placental mammals showing the exclusive presence of ZP3AR and ZFP819 in the Rodentia order. The most similar KZFP to ZFP819 present in other orders is ZNF510. ZNF510 and ZFP819 differ in their zinc finger domains. **(B)** Rodent family tree with those families containing ZP3AR and ZFP819 highlighted. **(C)** Rodent species tree with those species containing ZP3AR and ZFP819 indicated. Species are colored according to their rodent family. Selected nodes were represented on phylogenetic trees using the tool, http://www.timetree.org/. We used the database-integrated genome screening (DIGS) program (https://giffordlabcvr.github.io/DIGS-tool/) (and available at Zenodo: http://doi.org/10.5281/zenodo.6855611) to systematically screen mammalian genomes for sequences similar to *Zfp819* and ZP3AR.

Fig. S5

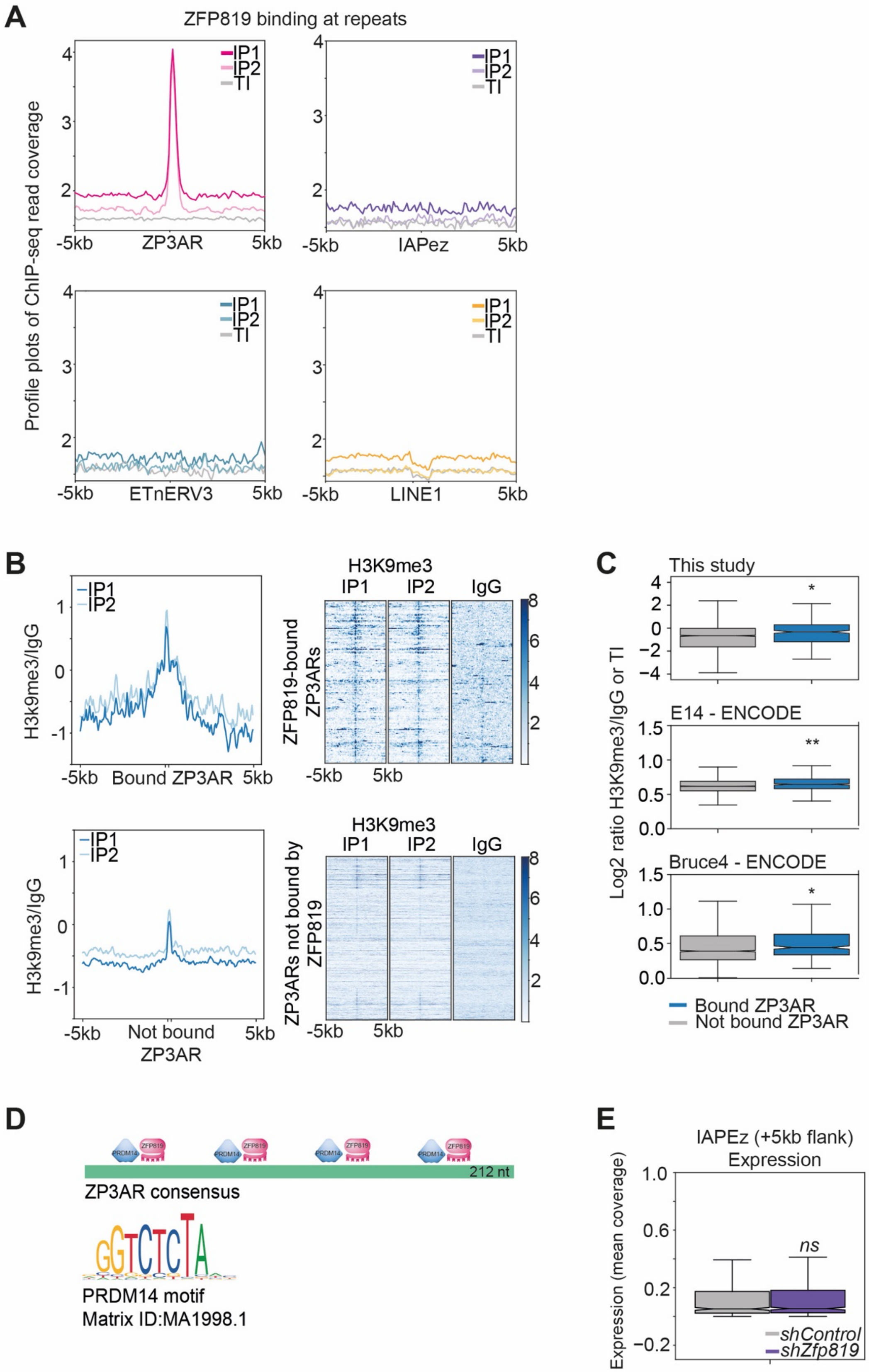
A
ZFP819 binding at repeats
Profile plots of ChIP-seq read coverage
IP1
IP2
TI
-5kb
ZP3AR
5kb
IAPez
ETnERV3
LINE1
B
H3K9me3/IgG
Bound ZP3AR
Not bound ZP3AR
H3K9me3
IP1
IP2
IgG
ZFP819-bound ZP3ARs
ZP3ARs not bound by ZFP819
C
This study
E14 - ENCODE
Bruce4 - ENCODE
Log2 ratio H3K9me3/IgG or TI
Bound ZP3AR
Not bound ZP3AR
D
PRDM14
ZFP819
212 nt
ZP3AR consensus
PRDM14 motif
Matrix ID:MA1998.1
E
IAPEz (+5kb flank)
Expression
Expression (mean coverage)
ns
shControl
shZfp819

Fig. S5. ZFP819-depletion induces a global erosion of H3K9me3.

(A) Profile plots show ZFP819-HA ChIP coverage signal at repeats depicted in Fig. 3a to illustrate that ZP3AR is the direct target. **(B)** H3K9me3 CUT&RUN coverage signal normalized to IgG control represented as profile plots and heatmaps, respectively, at ZFP819-HA-bound ZP3ARs (top) and unbound ZP3ARs (bottom) in mESCs. **(C)** Boxplot distribution of the mean H3K9me3 signal at ZP3ARs (with a 5kb flank) separated by ZFP819 binding (336 bound and 2239 unbound loci). Top: H3K9me3 CUT&RUN from this study (the data from A), *P-value* = 1.2180e-08. Middle: H3K9me3 ChIP in E14 mESCs, mouse ENCODE project, *P-value* = 0.0003. Bottom: H3K9me3 ChIP in Bruce4 mESCs, mouse ENCODE project, *P-value* = 0.0026. ENCODE data in mm10: E14 (ENCSR000ADM) - ENCFF286QJO (Rep 1) and ENCFF464DFI (Rep 2); Bruce4 (ENCSR000CFZ) - ENCFF284JNC (Rep 1) and ENCFF253DKG (Rep 2). TI: total input. **(D)** Enrichment of PRDM14 binding motif (JASPAR CORE database, MA1998.1), in reverse orientation, identified in ZP3AR sequences compared with shuffled sequences using the MEME suite tools (https://meme-suite.org/meme/). E-value = 1.98e-119 **(E)** Boxplot distribution of the mean IAPEz (599 full-length loci) expression signal (including the +/- 5kb flank) in *shControl* and *shZfp819* mESCs. The difference between groups is not significant (two-tailed paired t test, compared to *shControl*).

Fig. S6

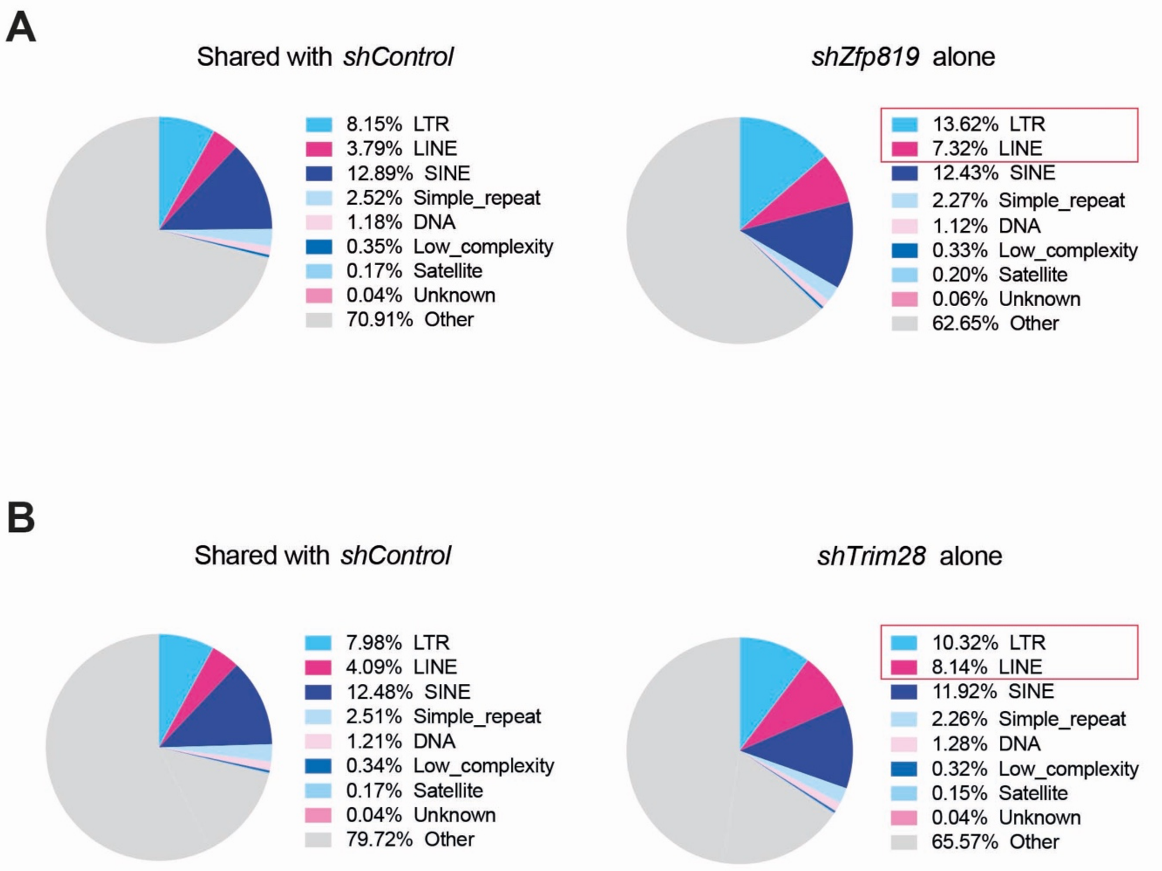
A
Shared with *shControl*
8.15% LTR
3.79% LINE
12.89% SINE
2.52% Simple_repeat
1.18% DNA
0.35% Low_complexity
0.17% Satellite
0.04% Unknown
70.91% Other
shZfp819 alone
13.62% LTR
7.32% LINE
12.43% SINE
2.27% Simple_repeat
1.12% DNA
0.33% Low_complexity
0.20% Satellite
0.06% Unknown
62.65% Other
B
Shared with *shControl*
7.98% LTR
4.09% LINE
12.48% SINE
2.51% Simple_repeat
1.21% DNA
0.34% Low_complexity
0.17% Satellite
0.04% Unknown
79.72% Other
shTrim28 alone
10.32% LTR
8.14% LINE
11.92% SINE
2.26% Simple_repeat
1.28% DNA
0.32% Low_complexity
0.15% Satellite
0.04% Unknown
65.57% Other

Fig. S6. ZFP819-depletion leads to an increase in global H3K27ac including at TE-derived enhancers

H3K27ac peaks were called in control mESCs (*shControl*) and in ZFP819- (*shRNA Zfp819 62*) and TRIM28-depleted mESCs. DiffBind was used to compare H3K27ac peaks between the control cells and the ZFP819-depleted cells and separately between the control cells and TRIM28-depleted cells. Pie charts represent a breakdown by percentage of the repeat classes overlapping shared H3K27ac peaks (left) and peaks unique to the ZFP819 or TRIM28-depleted cells (right) (*de novo* peaks). **(A)** Results for the comparison between control cells and ZFP819-depleted cells. **(B)** Results for the comparison between control cells and TRIM28-depleted cells. To assess which repeat classes were contributing to *de novo* peaks, the fold increase between the left and right-hand percentages were calculated for each repeat class. For both ZFP819-depleted cells and TRIM28-depleted cells, when looking at changes to whole repeat classes, there was only an increase in peaks overlapping LTRs and LINEs (shown by a pink box). Note that for ZFP819-bound ZP3ARs, they are already enriched in H3K27ac in control mESCs, where H3K9me3 is also present, the latter epigenetic mark which is lost in ZFP819-depleted cells. Repeats were ordered according to their fold increase of percentage in the ZFP819/TRIM28-depleted mESCs. See Fig. S7 for a breakdown of the top LTR and LINE hits.

Fig. S7

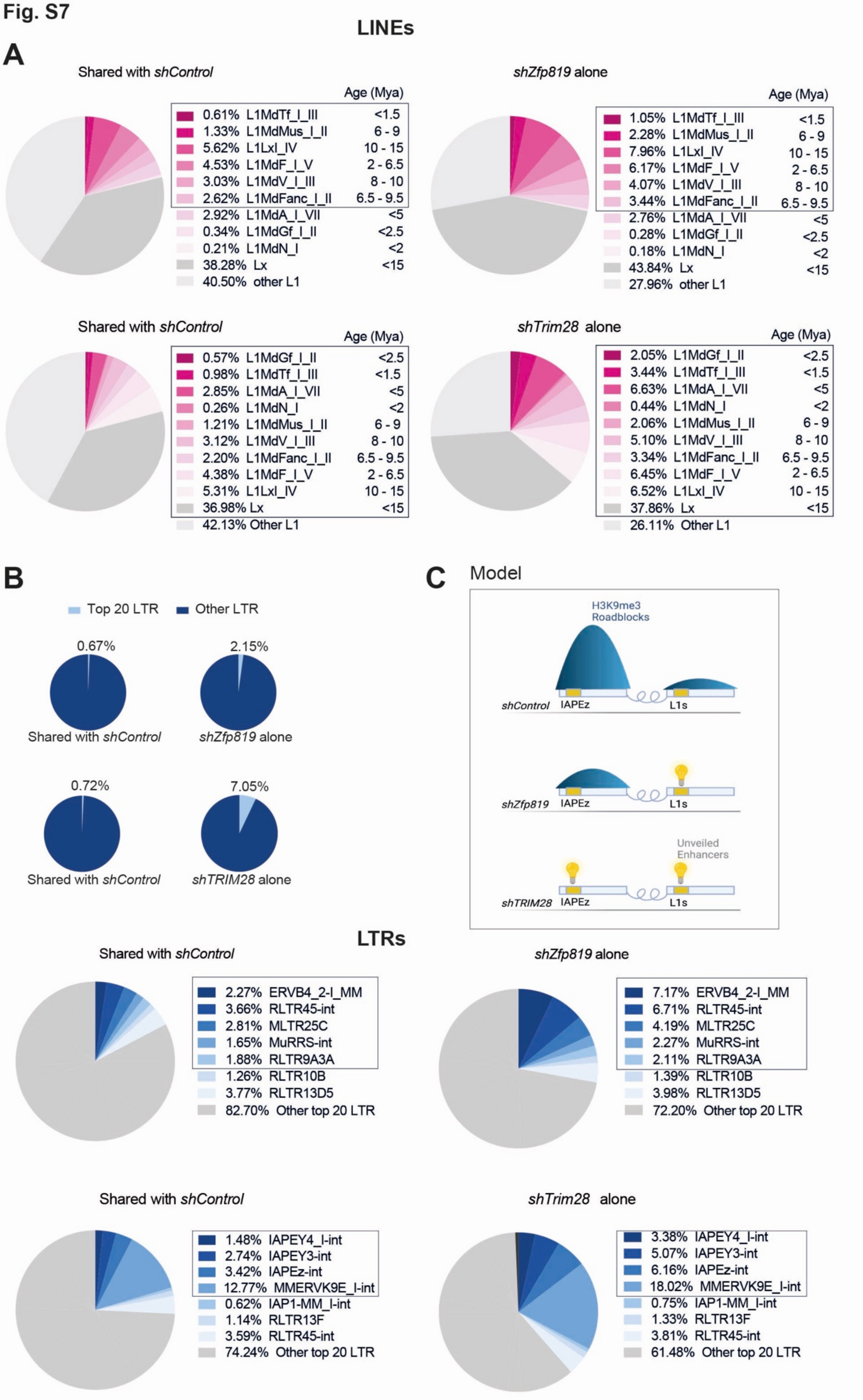
LINEs
A
Shared with shControl
Age (Mya)
0.61% L1MdTf_I_III <1.5
1.33% L1MdMus_I_II 6 - 9
5.62% L1LxI_IV 10 - 15
4.53% L1MdF_I_V 2 - 6.5
3.03% L1MdV_I_III 8 - 10
2.62% L1MdFanc_I_II 6.5 - 9.5
2.92% L1MdA_I_VII <5
0.34% L1MdGf_I_II <2.5
0.21% L1MdN_I <2
38.28% Lx <15
40.50% other L1
shZfp819 alone
Age (Mya)
1.05% L1MdTf_I_III <1.5
2.28% L1MdMus_I_II 6 - 9
7.96% L1LxI_IV 10 - 15
6.17% L1MdF_I_V 2 - 6.5
4.07% L1MdV_I_III 8 - 10
3.44% L1MdFanc_I_II 6.5 - 9.5
2.76% L1MdA_I_VII <5
0.28% L1MdGf_I_II <2.5
0.18% L1MdN_I <2
43.84% Lx <15
27.96% other L1
Shared with shControl
Age (Mya)
0.57% L1MdGf_I_II <2.5
0.98% L1MdTf_I_III <1.5
2.85% L1MdA_I_VII <5
0.26% L1MdN_I <2
1.21% L1MdMus_I_II 6 - 9
3.12% L1MdV_I_III 8 - 10
2.20% L1MdFanc_I_II 6.5 - 9.5
4.38% L1MdF_I_V 2 - 6.5
5.31% L1LxI_IV 10 - 15
36.98% Lx <15
42.13% Other L1
shTrim28 alone
Age (Mya)
2.05% L1MdGf_I_II <2.5
3.44% L1MdTf_I_III <1.5
6.63% L1MdA_I_VII <5
0.44% L1MdN_I <2
2.06% L1MdMus_I_II 6 - 9
5.10% L1MdV_I_III 8 - 10
3.34% L1MdFanc_I_II 6.5 - 9.5
6.45% L1MdF_I_V 2 - 6.5
6.52% L1LxI_IV 10 - 15
37.86% Lx <15
26.11% Other L1
B
Top 20 LTR
Other LTR
0.67%
Shared with shControl
2.15%
shZfp819 alone
0.72%
Shared with shControl
7.05%
shTRIM28 alone
C
Model
H3K9me3
Roadblocks
shControl
IAPEz
L1s
shZfp819
IAPEz
L1s
Unveiled
Enhancers
shTRIM28
IAPEz
L1s
LTRs
Shared with shControl
2.27% ERVB4_2-I_MM
3.66% RLTR45-int
2.81% MLTR25C
1.65% MuRRS-int
1.88% RLTR9A3A
1.26% RLTR10B
3.77% RLTR13D5
82.70% Other top 20 LTR
shZfp819 alone
7.17% ERVB4_2-I_MM
6.71% RLTR45-int
4.19% MLTR25C
2.27% MuRRS-int
2.11% RLTR9A3A
1.39% RLTR10B
3.98% RLTR13D5
72.20% Other top 20 LTR
Shared with shControl
1.48% IAPEY4_I-int
2.74% IAPEY3-int
3.42% IAPEz-int
12.77% MMERVK9E_I-int
0.62% IAP1-MM_I-int
1.14% RLTR13F
3.59% RLTR45-int
74.24% Other top 20 LTR
shTrim28 alone
3.38% IAPEY4_I-int
5.07% IAPEY3-int
6.16% IAPEz-int
18.02% MMERVK9E_I-int
0.75% IAP1-MM_I-int
1.33% RLTR13F
3.81% RLTR45-int
61.48% Other top 20 LTR

Fig. S7. ZFP819-depletion leads to an increase in H3K27ac at L1 elements and ERVs.
Data from Fig. S7. was used to investigate the increase in H3K27ac across specific TE subfamilies focusing on LTRs and LINEs (see Fig. S7). **(A)** A breakdown of all the LINE subfamilies by percentage is shown with the L1 categories shown in pink and the other LINEs in grey and grouped together. For *de novo* H3K27ac peaks occurring only in the ZFP819/TRIM28-depleted mESCs (right), young L1s (L1MdT) were most affected but also L1s up to 15 million years old. L1s are ordered by fold increase in percentage in the ZFP819/TRIM28-depleted mESCs. L1 ages are stated in million years. The L1 subfamilies that show an increase in the knockdown cells are boxed. *shRNA Zfp819 62* was used for ZFP819-depletion. **(B)** Percentage of overlap of H3K27ac peaks with LTRs (top four small pie charts). To give a breakdown of the LTR elements most represented in the *de novo* H3K27ac peaks, we focused on the top 20 families (bottom pie charts). LTR families were ordered by fold increase in percentage in the ZFP819/TRIM28-depleted mESCs. The boxes highlight LTR families displaying an increase in *de novo* enhancers. For ZFP819-depleted mESCs, ERVB4 LTRs were most affected, whereas for TRIM28-depleted cells, IAP elements were most affected. **(C)** Proposed model: In control mESCs, most H3K9me3 is concentrated at IAPEz elements (14fold enrichment, see Fig. 3). L1 elements have ~5x less H3K9me3 enrichment (2.6fold at L1MdA, see Fig. 3, and 1.8-2.4fold at L1MdTf elements, not shown). In ZFP819-depleted mESCs, in which heterochromatin roadblocks are eroded, enhancers at L1s are unveiled but not at IAPEz elements, which retain moderate H3K9me3. In TRIM28-depleted mESCs, heterochromatin roadblocks are lost and enhancers unveiled at both L1s and IAPEz elements.

Fig. S8

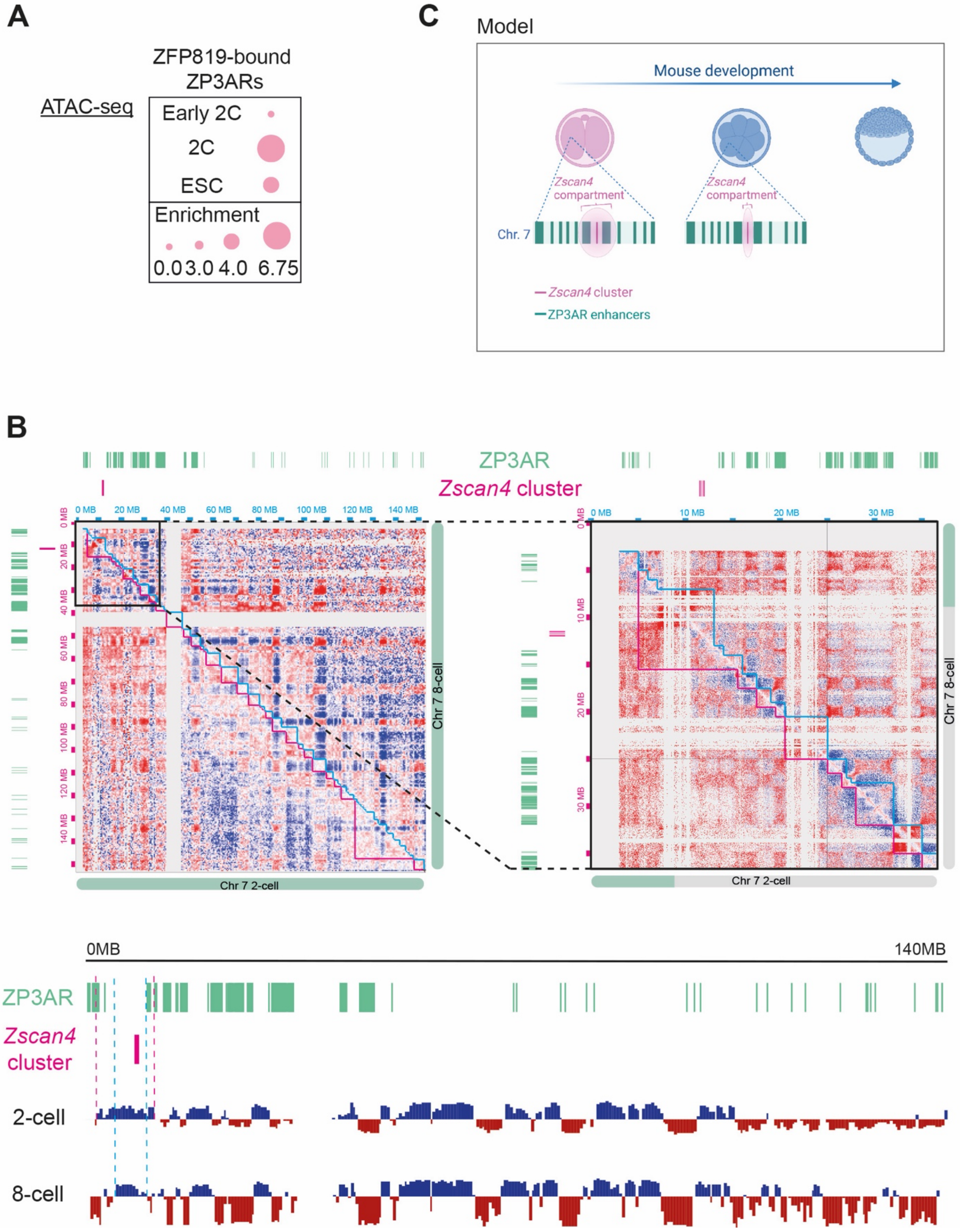
A
ZFP819-bound ZP3ARs
ATAC-seq
Early 2C
2C
ESC
Enrichment
0.0 3.0 4.0 6.75
C
Model
Mouse development
Zscan4 compartment
Zscan4 compartment
Chr. 7
Zscan4 cluster
ZP3AR enhancers
B
ZP3AR
Zscan4 cluster
Chr 7 8-cell
Chr 7 2-cell
Chr 7 8-cell
Chr 7 2-cell
0MB
140MB
ZP3AR
Zscan4 cluster
2-cell
8-cell

Fig. S8. Compartment switch between the 2-cell stage and 8-cell stage at the *Zscan4* gene cluster

(A) Observed over expected enrichment of chromatin accessibility as measured by ATAC-seq in early 2-cell, 2-cell and ESCs at ZFP819-HA-bound ZP3ARs, with enrichment values given. P-values were assessed using 1000 randomizations and were <0.05 for all stages, except the early 2-cells stage, which did not reach significance, likely due to the general accessibility of the genome at that stage (21). **(B)** Public dataset of HiC assessed at different stages of mouse development: Normalized observed over expected signal at chr7 at 500KB resolution (left) and 50KB resolution (right), after zooming in on the *Zscan4* gene cluster region. The upper diagonal shows interactions during the 8-cell stage of development and the lower diagonal shows interactions during the 2-cell stage. Compartment calls were derived from the first principal component (PC1, bottom) of the Pearson's correlation matrix for the observed over expected intra-chromosomal contacts at 500kb resolution using the KR normalization method. Pink lines across the heatmap depict compartments at the 2-cell stage. Blue lines across the heatmap depict compartments at the 8-cell stage. Compartments across the whole region at the 2-cell and 8-cell stage are also shown at the bottom with a switch shown as a blue to red shift. Note that compartments are not defined as either A or B type compartments here and compartment calls were defined using eigenvector values. ZP3AR and the *Zscan4* cluster are illustrated (61). **(C)** Model summary of the compartment switch at the *Zscan4* cluster: at the 2-cell stage, the *Zscan4* gene cluster has captured stretches of ZP3AR enhancers on either side of it within the same compartment. At the 8-cell stage, these ZP3AR enhancers have been excluded from the *Zscan4* compartment. Note that the ZP3AR barcode depicted here is not to scale in this model. Image created with BioRender.com.

Fig. S9

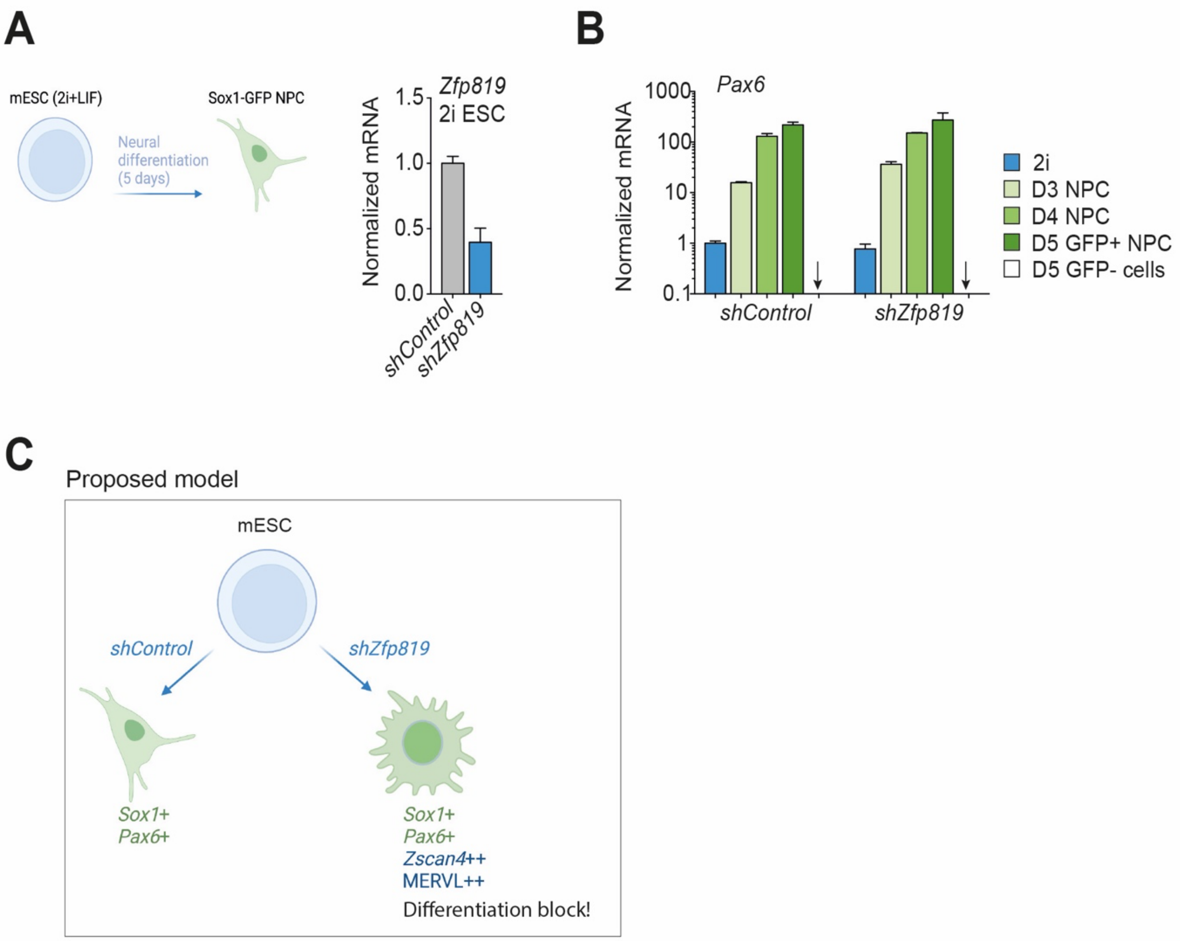
A
mESC (2i+LIF)
Sox1-GFP NPC
Neural differentiation (5 days)
Zfp819
2i ESC
Normalized mRNA
1.5
1.0
0.5
0.0
shControl
shZfp819
B
Pax6
Normalized mRNA
1000
100
10
1
0.1
shControl
shZfp819
2i
D3 NPC
D4 NPC
D5 GFP+ NPC
D5 GFP- cells
C
Proposed model
mESC
shControl
shZfp819
Sox1+
Pax6+
Sox1+
Pax6+
Zscan4++
MERVL++
Differentiation block!

Fig.S9. Unwarranted activation of the 2C program in ZFP819-depleted NPCs

(A) mESCs were cultured in 2i+LIF (naïve state) media and treated with *shControl* or *shZfp819* vectors before differentiation for 5 days to neural progenitor cells (NPCs). ZFP819-depletion was validated in 2i mESCs (right). Image created in BioRender.com. **(B)** RT-qPCR analysis of *Pax6* gene expression in Day 3, Day 4 and Day 5 sorted GFP+ NPCs. Expression was undetected in Day 5 sorted GFP- cells. Note that before sorting for GFP positive NPCs, there was a lower frequency of neural differentiation in ZFP819-depleted cells compared to the controls (see Fig.5A). Additionally, there was more cell death of ZFP819-depleted NPCs (Fig.5B). RT-qPCR validation of the 2C expressed genes, *Zscan4* and the endogenous retrovirus, MERVL upregulated in Day 3, Day 4 and Day 5 sorted GFP+ NPCs as well as in Day 5 sorted GFP- cells is shown in Fig. 5C. **(C)** Proposed model: The block in neural differentiation is likely not due to a failure to activate the neural transcriptional program, since SOX1-GFP protein and *Pax6* mRNA are expressed normally, but due to collateral effects of a failure to exit the 2C program. Unwarranted activation of *Zscan4* and MERVL likely affect differentiation potential. Image created in BioRender.com.

	Gene		Sequences
shRNA	*Zfp819*	*shZfp819 58*	CCGGGCGTGTTGTGTGCAGTTGTTTCTCGAGAA ACAACTGCACACAACACGCTTTTTG
		shZfp819 59	CCGGGCCAGTTGAATATACCAAATACTCGAGTAT TTGGTATATTCAACTGGCTTTTTG
		shZfp819 62	CCGGAGCCAGTTGAATATACCAAATCTCGAGATT TGGTATATTCAACTGGCTTTTTTG
	Trim28	*shTrim28*	CCGGGCTCTCTAAGAAGCTGATCTACTCGAGTA GATCAGCTTCTTAGAGAGCTTTTTG
qRT-PCR Primers	*Zscan4*	For	CAAGGCCAACACCAGATAATG
		Rev	TCCACTACAGCTTTCACCAAC
	MERVL	For	GGCCTATTTACCAAGTGACTCG
		Rev	CAGACACCTCAAGTACCATCG
	Chit1	For	TGGGCAGGTGTGATGACTCT
		Rev	CCCTGGGAAAGAACCGAACTG
	Usp17l	For	GAGGTCTTTGGAGACATGGTG
		Rev	CCAACTCAGACTGTGCTTTCC
	Zfp819	For	GACGTGTTGCTGGAGATCTAC
		Rev	AATCAACCTTCCCCATCGG
	Trim28	For	CGGAAATGTGAGCGTGTTCTC
		Rev	CGGTAGCCAGCTGATGCAA
	Gapdh	For	TCCATGACAACTTTGGCATTG
		Rev	CAGTCTTCTGGGTGGCAGTGA
	Cox6a1	For	CTCTTCCACAACCCTCATGT
		Rev	GAGGCCAGGTTCTCTTTAC
	Pou5f1	For	GATGCTGTGAGCCAAGGCAAG
		Rev	GGCTCCTGATCAACAGCATCAC
ChIP-/CUT&RUN-PCR Primers	*Gnas*	For	TGCCCAGGAATAATCTGCAGA
		Rev	ATACAGTCACATTGCCCGGT
	Ccna2	For	AGTAGCCCGCGACTATTGAAAT
		Rev	GCGACCGGCGCTTCT
	Pou5f1 Enh	For	GGAGGCAAGAAACTGGATCAGA
		Rev	CCTGGTGGCCCTGGAGAT
	Gapdh	For	GGCCGCCGCCATGT
		Rev	AGCTAGGAAGAAGGAAGGCCTAAG
	Zfp180	For	CCGTACAGGTGCAATCTGTG
		Rev	GTTTGTAGCTCTGGCGGAAC

Table S1.

shRNA and primer sequences

Protein	Species from	Cat. No.	Manufacturer
PCNA	Mouse	NA03	Calbiochem
ZSCAN4	Rabbit	AB4340	Millipore
SETDB1	Rabbit	11231-1-AP	Proteintech
POU5F1	Mouse	sc-5279	Santa Cruz Biotechnology
TRIM28	Rabbit	ab10483	Abcam
HA	Rabbit	12013819001	Roche
HA (ChIP)	Mouse	901501	Biolegend
H3K9me3	Rabbit	ab8898	Abcam
H3K27ac	Rabbit	ab4729	Abcam
H3	Rabbit	ab21054	Abcam
IgG	Rabbit	12-370	Millipore

Table S2.
Antibodies

Data file S1. (separate file)

Supplementary Data file S1: Differentially expressed genes and repeats
